# Single-Cell-Based Analysis Highlights a Surge in Cell-to-Cell Molecular Variability Preceding Irreversible Commitment in a Differentiation Process

**DOI:** 10.1371/journal.pbio.1002585

**Published:** 2016-12-27

**Authors:** Angélique Richard, Loïs Boullu, Ulysse Herbach, Arnaud Bonnafoux, Valérie Morin, Elodie Vallin, Anissa Guillemin, Nan Papili Gao, Rudiyanto Gunawan, Jérémie Cosette, Ophélie Arnaud, Jean-Jacques Kupiec, Thibault Espinasse, Sandrine Gonin-Giraud, Olivier Gandrillon

**Affiliations:** 1 Univ Lyon, ENS de Lyon, Univ Claude Bernard, CNRS UMR 5239, INSERM U1210, Laboratory of Biology and Modelling of the Cell, 46 allée d’Italie Site Jacques Monod, F-69007, Lyon, France; 2 Inria Team Dracula, Inria Center Grenoble Rhône-Alpes, France; 3 Université de Lyon, Université Lyon 1, CNRS UMR 5208, Institut Camille Jordan 43 blvd du 11 novembre 1918, F-69622 Villeurbanne-Cedex, France; 4 Département de Mathématiques et de statistiques de l’Université de Montréal, Pavillon André-Aisenstadt, 2920, chemin de la Tour, Montréal (Québec) H3T 1J4 Canada; 5 The CoSMo company. 5 passage du Vercors – 69007 LYON – France; 6 Univ Lyon, Univ Claude Bernard, CNRS UMR 5310 - INSERM U1217, Institut NeuroMyoGène, F-69622 Villeurbanne-Cedex, France; 7 Institute for Chemical and Bioengineering, ETH Zurich, Zurich, Switzerland; 8 Swiss Institute of Bioinformatics, Quartier Sorge - Batiment Genopode, 1015 Lausanne Switzerland; 9 Genethon – Institut National de la Santé et de la Recherche Médicale – INSERM, Université d’Evry-Val-d’Essone – 1 rue de l’internationale 91000 Evry, France; 10 RIKEN - Center for Life Science Technologies (Division of Genomic Technologies)—CLST (DGT), 1-7-22 Suehiro-cho, Tsurumi-ku, Yokohama, Kanagawa 230-0045, Japan; 11 INSERM, Centre Cavaillès, Ecole Normale Supérieure, F-75005 Paris, France; EMBL-European Bioinformatics Institute & Wellcome Trust Sanger Institute, UNITED KINGDOM

## Abstract

In some recent studies, a view emerged that stochastic dynamics governing the switching of cells from one differentiation state to another could be characterized by a peak in gene expression variability at the point of fate commitment. We have tested this hypothesis at the single-cell level by analyzing primary chicken erythroid progenitors through their differentiation process and measuring the expression of selected genes at six sequential time-points after induction of differentiation. In contrast to population-based expression data, single-cell gene expression data revealed a high cell-to-cell variability, which was masked by averaging. We were able to show that the correlation network was a very dynamical entity and that a subgroup of genes tend to follow the predictions from the dynamical network biomarker (DNB) theory. In addition, we also identified a small group of functionally related genes encoding proteins involved in sterol synthesis that could act as the initial drivers of the differentiation. In order to assess quantitatively the cell-to-cell variability in gene expression and its evolution in time, we used Shannon entropy as a measure of the heterogeneity. Entropy values showed a significant increase in the first 8 h of the differentiation process, reaching a peak between 8 and 24 h, before decreasing to significantly lower values. Moreover, we observed that the previous point of maximum entropy precedes two paramount key points: an irreversible commitment to differentiation between 24 and 48 h followed by a significant increase in cell size variability at 48 h. In conclusion, when analyzed at the single cell level, the differentiation process looks very different from its classical population average view. New observables (like entropy) can be computed, the behavior of which is fully compatible with the idea that differentiation is not a “simple” program that all cells execute identically but results from the dynamical behavior of the underlying molecular network.

## Abstract 2

A single-cell transcriptomics analysis offers a new dynamical view of the differentiation process, involving an increase in between-cell variability prior to commitment.

## Author Summary

The differentiation process has classically been seen as a stereotyped program leading from one progenitor toward a functional cell. This vision was based upon cell population-based analyses averaged over millions of cells. However, new methods have recently emerged that allow interrogation of the molecular content at the single-cell level, challenging this view with a new model suggesting that cell-to-cell gene expression stochasticity could play a key role in differentiation. We took advantage of a physiologically relevant avian cellular model to analyze the expression level of 92 genes in individual cells collected at several time-points during differentiation. We first observed that the process analyzed at the single-cell level is very different and much less well ordered than the population-based average view. Furthermore, we showed that cell-to-cell variability in gene expression peaks transiently before strongly decreasing. This rise in variability precedes two key events: an irreversible commitment to differentiation, followed by a significant increase in cell size variability. Altogether, our results support the idea that differentiation is not a “simple” series of well-ordered molecular events executed identically by all cells in a population but likely results from dynamical behavior of the underlying molecular network.

## Introduction

The classical view of a linear differentiation process driven by the sequential activation of master regulators [1] has been increasingly challenged in the last few years both by experimental findings and theoretical considerations.

Thanks to the recent development in single-cell profiling technologies, researchers are now able to investigate qualitatively and quantitatively the cell-to-cell variability in gene expression in more detail. In this context, several experimental studies at single-cell level involving the regulation of self-renewal and differentiation processes in embryonic stem cells [2–8] and the generation of induced pluripotent stem cells [9] have shown that gene expression variability might be involved in cell differentiation. To support this claim, recent researches on hematopoietic stem cells highlighted the role of molecular heterogeneity in differentiation [10, 11]. Further evidence was also obtained during an ex vivo differentiation process [12], and in the generation of cells of the immune system [13–18].

The overt cell-to-cell variability is deeply rooted in the inherent stochasticity of the gene expression process [19–23]. Numerous explanations have been put forward regarding the molecular and cellular sources for such variability (see [24] and references therein). Some of those causes involve biophysical processes (e.g., the random partitioning during mitosis, as discussed in [25]), whereas others are more related to biochemical regulation (e.g., the dynamical functioning of the intracellular network [26] or the chromatin dynamics [27]).

At least three models of cell differentiation based on stochastic gene expression have been proposed, in which a peak in the gene expression variability is expected to occur. In the first model, stochastic gene expression is the driving force of cell differentiation that generates cell type diversity, on which a selective constraint is then exerted [28]. In the second model, noise in gene expression causes bifurcations in the dynamics of gene regulatory networks [21]. In the third model, cell differentiation is viewed as a dynamical process in which differentiating cells are thought of as particles moving around in a state space [29, 30]. This formal space can be used to display gene expression patterns. Hence, when some parameters that describe gene regulatory interactions change, the cell particle “moves” in the state space. In this view, discrete identified cell states (e.g., self-renewing, differentiated) correspond to different regions of this space that could be seen as different attractor states. The transition process between attractors therefore first requires the exit from the original state that may be fueled by an increase in gene expression stochasticity [31]. Regardless of the differences between these models, they all assume that the differentiation process is represented by cell trajectories leading from one state to another through a phase of biased random walk in gene expression. This phase is followed by stabilization (convergence) toward a particular pattern of gene expression corresponding to a stable attractor state, the differentiated final state, in which noisy fluctuations of gene expression is minimized by the stabilizing effect of the attractor. Therefore, changes in the extent of cell-cell variability could be a new observable metric to characterize the cell differentiation process.

The purpose of the present study was then to assess whether gene expression variability changes during the differentiation process, as suggested by the above-quoted models, and whether such variation concurs with any physiological cellular change. We investigated the extent of gene expression variability at the single-cell level, both before and during the cell differentiation process. To do this, we analyzed the differentiation process of T2EC, which is an original cellular system consisting of non-genetically modified avian erythrocytic progenitor cells grown from a primary culture [32]. These cells can be maintained ex vivo in a self-renewal state under a combination of growth factors (TGF-*α*, TGF-*β*, and dexamethasone) and can also be induced to differentiate exclusively toward erythrocytes by changing the combination of the external factors present in the medium. The primary cause for differentiation is therefore known and relies upon change in the information carried by the extracellular environment. The differentiation process in those cells has been previously analyzed at the population level [33–35].

We first selected a pool of 110 relevant genes on the basis of RNA-Seq analysis performed on populations of T2EC in self-renewal state or induced to differentiate for 48 h. Multivariate statistical analysis of the data allowed us to select 92 genes for further analysis. We then performed high-throughput reverse transcription followed by reverse transcription quantitative PCR (RT-qPCR) of the 92 selected genes on single-cells collected at six time-points of differentiation. Several dimensionality reduction algorithms were used to visualize trends in the datasets. In agreement with the above hypothesis, cell heterogeneity, as measured by entropy, significantly increased during the first hours of the differentiation process and reached a maximal value at 8 to 24 h before decreasing toward the end of the process. The peak in entropy preceded an increase in cell size variability at 48 h. These observations suggested that 24 h is a crucial turning point in the erythrocytic differentiation process, which was experimentally verified by showing that T2EC committed irreversibly to the differentiation process between 24 h and 48 h.

## Results

### Identification of Differentially Expressed Genes Between Self-Renewing and Differentiating Progenitors

In order to identify a pool of genes potentially relevant in the differentiation process, we analyzed the transcriptome of self-renewing and differentiating primary chicken erythrocytic progenitor cells (T2EC) using RNA-Seq. We sequenced two independent libraries from self-renewing T2EC and two independent libraries from T2EC induced to differentiate for 48 h. For each condition, we first verified that read counts between replicates were reproducible (S3A and S3B Fig). We then identified 424 significantly differentially expressed genes (*p*-value < 0.05, S3C Fig). Gene ontology analysis using the DAVID database [60] revealed a clear over-representation of genes involved in sterol biosynthesis in this list (not shown). This finding was in line with our previous analysis showing that the oxydosqualene cyclase (OSC), which is involved in cholesterol synthesis, is required to maintain self-renewal in T2EC [35]. However, no other over-represented function emerged from the present analysis.

### Identification of Genes Relevant to Analyze the Erythrocytic Differentiation Process

To identify a smaller subset of relevant genes for further analysis by RT-qPCR using the Fluidigm array (see below), we tested 56 down-regulated and 77 up-regulated genes among the above 424 genes differentially expressed in self-renewing versus differentiating cells, which had the smallest set of *p*-values. We also included 32 non-regulated genes, selected among the most invariant ones. We then measured the expression of these 165 genes first using RNA from bulk cell populations taken at five time-points during differentiation (0, 8, 24, 48, and 72 h). Based on qPCR primer efficiency, 55 genes were removed (see Materials and Methods), which left a total of 110 genes for the subsequent analysis.

**Fig 1:**
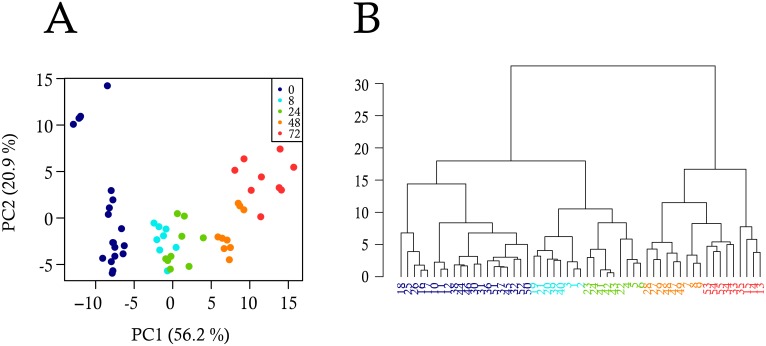
Analysis of bulk-cell gene expression during the differentiation process. Gene expression data were produced by RT-qPCR in triplicate from three independent T2EC populations collected at five differentiation time-points (0 h, 8 h, 24 h, 48 h, 72 h). The expression level of 110 genes (18 invariants, 50 down-regulated and 42 up-regulated) was analyzed by two different multivariate statistical methods: (A) Principal component analysis (PCA), and (B) Dendogram resulting from hierarchical cluster analysis (HCA). The dots in (A) and leaves in (B) indicate the different cell populations and the colors indicate the differentiation time-points at which they were collected.

A principal component analysis (PCA) on the bulk gene expression levels (Fig 1A) showed a clear separation of the time-point 0 h (self-renewal) from the differentiation time-points. Samples along the differentiation process were well ordered according to the first principal component (PC1). PC1 explained 56.2% of the data variability suggesting that the differentiation process is the main source of variability at the population level for the selected genes.

We also performed a hierarchical cluster analysis (HCA), which again showed a clear arrangement of the samples according to their position along the differentiation process (Fig 1B). We further noticed that the gene expression patterns at 0, 8, and 24 h time-points were more similar to each other, while those at 48 h and 72 h time-points were also more similar to each other.

Thus, the 110 selected genes allowed us to clearly distinguish cell populations according to their progression along the differentiation sequence, indicating that they were relevant for analyzing this process. However, since the single-cell measurement technology used in this study could only accommodate 92 genes (not including two spikes and two repeats for the *RPL22L1* gene), we further refined our gene choice by performing a K-means clustering on the above data. The algorithm grouped genes based on their expression profile, and identified seven different gene clusters with respect to expression kinetics (S4 Fig).

The patterns mainly showed decreasing or increasing gene expressions during the differentiation process, while one cluster displayed a more complex dynamic (cluster 4). The latter was composed of genes whose expression decreased during the first 8 h, then increased and stabilized between 24 h and 48 h, before decreasing again until 72 h. Interestingly, all genes belonging to this cluster were linked by their involvement in sterol biosynthesis, reinforcing the previously noted role of this pathway in erythroid differentiation. Based on the result of K-means clustering, we selected around thirteen genes per group to represent each cluster equally. This left us with 92 genes for further analysis (S1 Table).

We then used STRING database to search for known connections among these genes. The result confirmed the existence of a strongly connected subnetwork associated with sterol synthesis (S5B Fig). Moreover, this analysis also revealed the presence of another highly connected subnetwork mostly composed of genes involved in signaling cascades and two transcription factors (BATF and RUNX2). Those two main networks are linked by the gene *HSP90AA1* which encodes the molecular chaperone HSP90*alpha*. Its activity is not only involved in stress response but also in many different molecular and biological processes because of its important interactome. HSP90*alpha* represents 1%–2% of total cellular protein in unstressed cells. Interestingly, HSP90*alpha* level is up-regulated and correlated with poor disease prognosis in leukemia [61]. HSP90*alpha* has also been shown to be involved in the survival of cancer cells in hypoxic conditions [62].

### Cell-to-Cell Heterogeneity Blurred Cell Differentiation Process

We measured the expression level of the selected 92 genes by single-cell RT-qPCR using 96 cells isolated from the most informative time-points of the differentiation sequence. Based upon preliminary experiments, we decided to analyze cells from six time-points during differentiation. After data cleaning (see Materials and Methods), we obtained the expression level of 90 genes in 55, 73, 72, 70, 68, and 51 single cells from 0, 8, 24, 33, 48, and 72 h of differentiation, respectively.

One should note that the variability we observed at the single-cell level originates from two types of sources: biological sources and experimental sources. We therefore tested the technical reproducibility of different RT-qPCR steps liable to generate such experimental noise (see Materials and Methods). As expected, reverse transcription (RT) was the main source of experimental variability, since pre-amplification and qPCR steps brought negligible amount of variability (S1 Fig). Moreover, using external RNA spikes controls whose Cq value depends only on the experimental procedure, we noted that technical variability was negligible compared to the biological variability (see Materials and Methods). Quality control (see Materials and Methods) led to the elimination of 2 genes, letting us with 90 genes for subsequent analysis.

**Fig 2:**
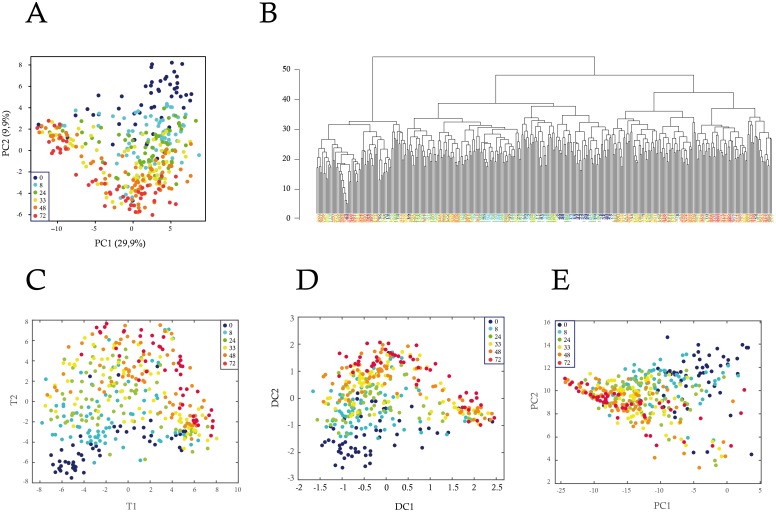
Analysis of single-cell gene expression during the differentiation process. Gene expression data were produced by RT-qPCR from individual T2EC collected at six differentiation time-points (0, 8, 24, 33, 48, and 72 h). The expression of 90 genes was analyzed in single-cells by five different multivariate statistical methods: (A) Principal component analysis (PCA), (B) Hierarchical cluster analysis (HCA), (C) t-SNE, (D) Diffusion map, and (E) kernel PCA. The dots in (A, C, D, and E) and leaves in (B) indicate the single-cells, and the colors indicate the differentiation time-points at which they were collected. t-SNE analysis was performed using the following parameters: initial_dims = 30; perplexity = 60. Diffusion map was run using the following parameters: no_dims = 4, t = 1, and sigma = 1000. Kernel PCA was run with a parameter for computing the “poly” and “gaussian” kernel of 0.1. Only the first two dimensions are plotted.

We first used PCA on the single-cell expression of these 90 genes (Fig 2A). In contrast to the whole-population data, the single-cell data did not immediately demarcate into well-separated clusters. The differentiation process was most apparent by looking at the second principal component (PC2), which explained 9.9% of the variability in the dataset. Hence, unlike in the population-averaged data, the differentiation process did not represent the main source of variability at the single-cell level.

The application of HCA further confirmed that the classification became more complex for single-cell data (Fig 2B). Contrary to bulk analysis, individual cells from the same time-point were not necessarily more similar to each other than to cells from neighboring time-points. Consequently, the clustering of individual cells into groups became complicated. The picture of cell differentiation process that emerged from the single-cell analysis thus far was more complex than the one obtained from the population level analysis. This difference between single-cell and population-level analysis arises from the unraveling of cell-to-cell heterogeneity in the single-cell data, which could have been hidden by the averaging effect of the population (see below).

PCA is a linear method for dimensionality reduction of single-cell data. In view of non-linear relationships of cell states in state space, recently nonlinear techniques like t-SNE [55] or diffusion maps [63] have been applied in single-cell data analysis. t-SNE is a variation of Stochastic Neighbor Embedding deemed capable of capturing more local structures than classical PCA, while also revealing global structure such as the presence of clusters at several scales. Diffusion maps use a non-linear distance metric (referred to as diffusion distance), which is deemed conceptually relevant in view of noisy diffusion-like dynamics during differentiation [63]. We therefore applied these algorithms on our datasets, as well as another non-linear version of PCA, called Kernel PCA [64], not previously applied to single-cell gene expression data (Fig 2C to 2E). The general conclusions obtained by PCA did not appreciably change when using these non-linear dimensionality reduction techniques. There was again an obvious trend reflecting the differentiation process, as well as a significant amount of intermingling of cells from different time-points.

### Single-Cell Data Embed Population Information and Reveal New Discriminating Genes Involved in the Differentiation Process

**Fig 3:**
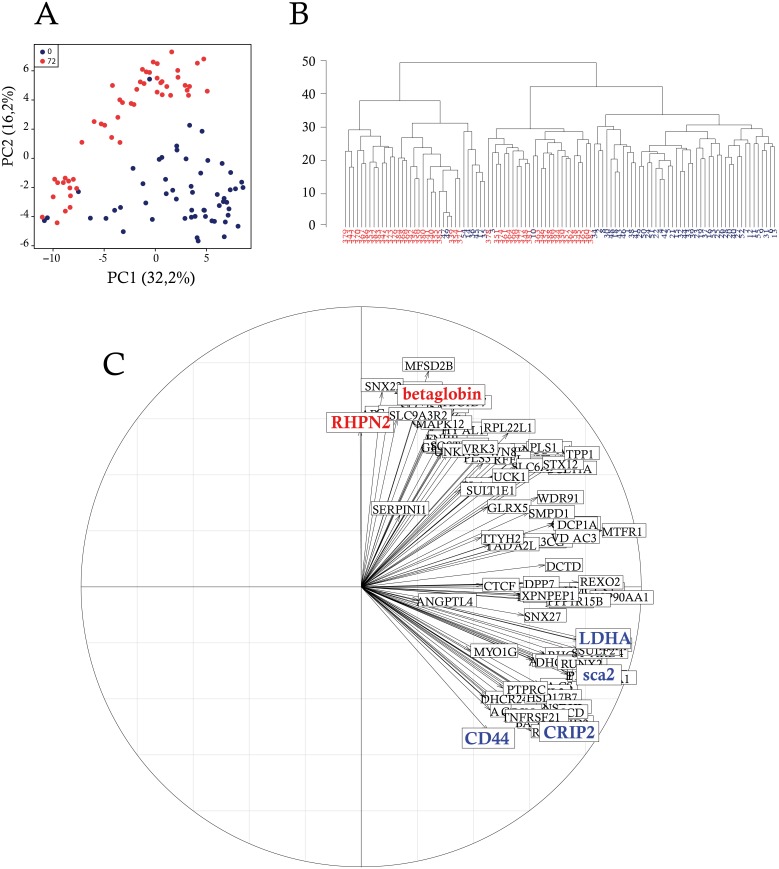
Gene expression-based discrimination between self-renewing and differentiating individual cells. Single-cell gene expression data were analyzed considering only self-renewing cells and cells induced to differentiate since 72 h. (A) Principal component analysis (PCA); (B) Hierarchical cluster analysis (HCA) was used to sort single-cells picked up at 0 h and 72 h of the differentiation process according to similarity measurement; (C) Two-dimensional representation of the contribution of each variable (gene) to the inertia. The direction of the arrows displays the contribution of that variable to the underlying component. The colored genes highlight genes of interest and genes that contributed the most to the PCA outcome, associated with self-renewal (blue) and the erythroid differentiation process (red).

In order to assess to what extent the differentiation process was still visible in the single-cell data, we performed PCA on datasets from the two extreme time-points, 0 and 72 h (Fig 3A). The result showed a clear separation of both time-points with only a few cells intermingled. We also performed HCA on datasets from the same time-points (Fig 3B). Again, the segregation of the cells was still not perfect, but cells were not as mixed as before. Here, there exist two clusters of self-renewing and differentiating cells. When compared to the analysis of the entire time series, the separation between cells from the two extreme time-points looked clearer. Therefore, the analysis of single-cell data confirmed that part of the information present in the single-cell data is linked to the differentiation process.

The idea that shared information was present in single-cell and population-based data was reinforced by the analysis of the correlation matrices within and between the two datasets (S6 Fig). It was apparent that (1) the global intensity of the correlations was higher with population-based data and (2) there existed a co-structure between the two datasets. At the population level, we showed that the set of genes selected was relevant to analyze the differentiation process (Fig 1). The cross-correlation analysis strengthened this view and demonstrated that when looking at the single-cell scale, the information held by these genes was not totally erased by cell-to-cell variability.

We then looked at the genes that contributed the most to the PCA outcome (Fig 3C). Among the genes that discriminate the most self-renewing cells, one could highlight *LDHA* (Lactate deshydrogenase A), *CRIP2*, and *Sca2*. *Sca2* is a gene that we previously have shown to be associated with the self-renewal of erythroid progenitors [34]. *LDHA* is less expected and will be discussed below. Among the genes that contributed the most to discriminating differentiated cells, one could highlight *RHPN2* and *betaglobin*. Since betaglobin is a part of hemoglobin, the most abundant protein in erythrocytes, it was expected to be associated with differentiating cells.

### Single-Cell Data Averaging Recapitulates Results from Population-Level Analysis

**Fig 4:**
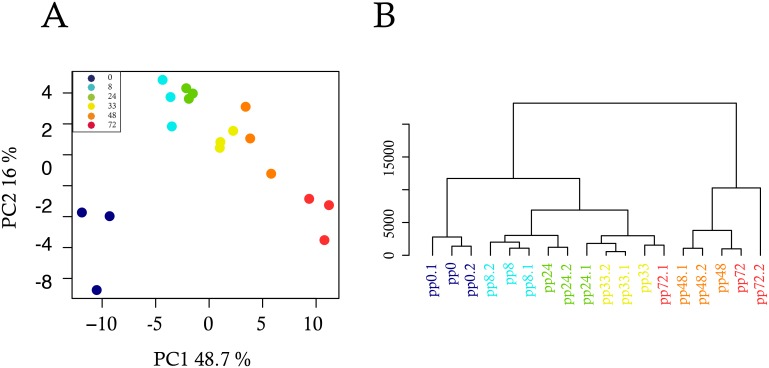
Analysis of single-cell data averaged over pseudo-populations. We separated single-cells into three pseudo-populations with around one-third of single cells for each time-point. We then calculated the average gene expression over each pseudo-population, and analyzed the resulting averaged data using multivariate statistical methods. (A) Principal component analysis (PCA); (B) Hierarchical cluster analysis (HCA).

Given that the analysis of single-cell gene expression did not produce a clear separation of the temporal stages, in contrast to whole populations, we hypothesized that by averaging over a population of individual cells, we should be able to reproduce the bulk results. For this purpose, we generated three pseudo-populations (sub-populations) of about one-third of cells from the single-cell data and computed their average gene expressions for each time-point. By performing PCA on the mean gene expressions of these pseudo-populations, we noticed that the averaged data showed more organization and, importantly, that the differentiation progression materialized along the PC1 dimension (Fig 4A).

The PCA result of the pseudo-population therefore looked much more like the population than the single-cell results. Similarly, HCA generated a clustering that was not quite as clear as the analysis of bulk RNA data, but much better than the single-cell analysis (Fig 4B). The HCA results showed for example similarities between gene expressions from time-points 48 and 72 h. Together the pseudo-population analysis obtained by statistical averaging of single-cell data mostly recapitulated, albeit not entirely, the population-based results, suggesting that the clear-cut classification of bulk-cell-based data is due to the (physical) averaging effect in populations, in line with a previous account [65].

### The Correlation Networks are Very Dynamical Entities

Single-cell data offers access to the patterns of the relationship of genes with respect to both their marginal (S7 Fig), as well as their full joint distribution (not shown). This provides us with a new observable that we used to characterize the progression of the differentiation process in finer details.

For each time-point, we computed a correlation matrix to evaluate how correlated the expression of any pair of genes was, across all cells at a given time. Since data were log-normally distributed, we employed the Spearman correlation coefficient. We then calculated the significance of the correlation and used a *p*-value below 0.05 as a cutoff. Two genes (the nodes of a graph) that exhibited a significant correlation were connected by an edge. Finally, we sub-sampled 85% of the cells for 10,000 iterations, so as to obtain robust correlation networks that will not depend upon the sampling process. We then constructed a gene correlation network for each time-point. Although both positive and negative correlations were computed, negative correlations proved much less robust and were eliminated by the sub-sampling process, in which we only kept significant correlations that appeared in all of the 10,000 subsampling.

**Fig 5:**
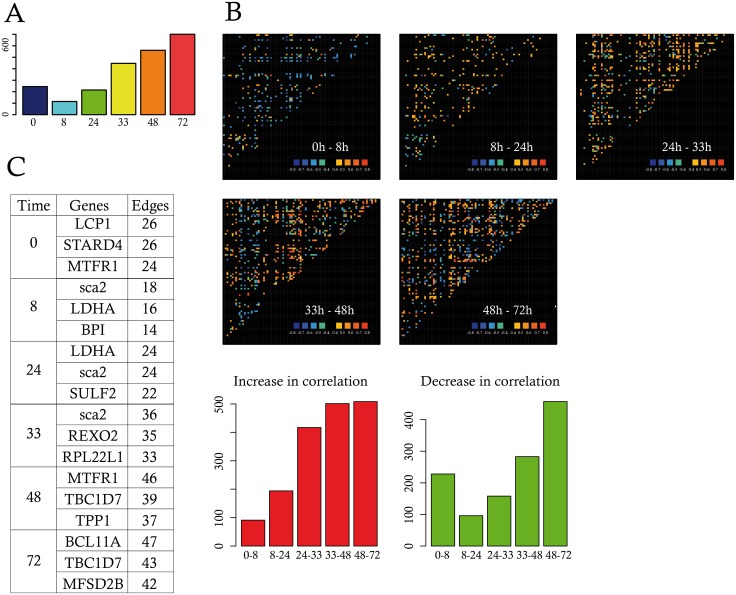
Gene expression correlations. (A) Shown is the number of significant correlations, between any pair of genes, surviving 10,000 sub-sampling iterations, per time-point; (B) Correlation variations between two consecutive time-points using the color code bar shown at the bottom right of the panels. Cold colors (blue and green) indicate decreasing genes correlations and hot colors (from yellow to red) stand for increasing gene correlations between the time-points considered. Intermediary variations (between −0.4 and +0.4) as displayed in black. The bottom left red barplot indicates the number of increasing correlations, whereas the green barplot shows the number of decreasing correlations between each pair of consecutive time-points; (C) The three genes that displayed the highest number of edges at each time-point were listed in the table, as well as the number of edges connecting those genes. Data for this figure (A and B) can be found at osf.io/k2q5b.

As shown in (Fig 5A), the density of the resulting networks (number of significant correlations) was clearly varying along the differentiation process.

One observed a sudden drop in the number of correlations by 8 h that then steadily increased to reach a maximum value at 72 h much higher than the initial value. Interestingly, this global behavior resulted from both an increase and a decrease in gene-to-gene correlation values (Fig 5B). Even between 48 and 72 h, some gene pair correlation decreased while the overall net balance resulted in a global increase.

This fast-changing density of the networks was also accompanied by a progressive change in the identity of the most highly correlated nodes (Fig 5C). Both *Sca2* and *LDHA* that were previously identified by the PCA also appeared as prominent among the correlation network from 8 to 24 h, while later time-points were characterized by the appearance of other genes as *TBC1D7* and *BCL11A*.

One should note that such correlation networks are to be seen as resulting from the behavior of the underlying mechanistic gene interaction networks, but can not be taken per se as a faithful representation of such dynamical interaction networks.

### Evidence for the DNB Theory

**Fig 6:**
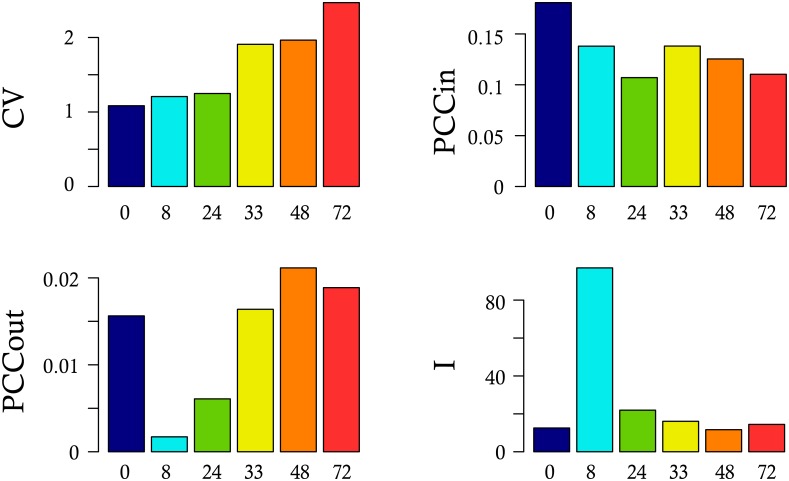
Identification of a dynamical network biomarker. Shown is the behavior of a subset composed of 12 genes fitting the following criteria: increase in their standard deviation and participation to increasing correlations, between 0h and 8h. For this subset, we plotted the mean coefficent of variation (CV), the mean of the correlation between any pair of genes belonging to the subset (PCCin), the mean of the correlation between any one gene of the subset and any one gene outside of the subset (PCCout) and the resulting I-scores, at each time-point. The DNB group included the following genes: *ACSS1*, *ALAS1*, *BATF*, *BPI*, *CD151*, *CRIP2*, *DCP1A*, *EMB*, *FHL3*, *HSP90AA1*, *LCP1*, *MTFR1*. Data for this figure can be found at osf.io/k2q5b.

Contrary to previous accounts [12, 66], we observed a global decrease in the correlation intensity between 0 and 8 h. Nevertheless, we noticed that some gene pairs showed an increased correlation coefficient. We therefore reasoned that those genes could represent a putative dynamical network biomarker (DNB), a subgroup of genes involved in the critical transition phase of a dynamical system [51]. To qualify for a DNB, three conditions have to be fulfilled: (1) the coefficent of variation (CV) of each variable in the DNB should increase, (2) the correlation (PCCin) within the DNB should increase, and (3) the correlation (PCCout) between the DNB and outside genes should decrease. All three conditions can be simultaneously quantified using the I score (see Materials and Methods). We therefore first selected a group of 12 genes by a two-stage process: (1) we first selected all of the genes that participated in at least one pair that showed an increased correlation of at least 0.5 between 0 and 8 h and (2) among those genes, we selected the genes that showed an increase in their CV value between 0 and 8 h. We then computed the I score of that group of genes at each time-point (Fig 6).

Although PCCin slightly decreased with time, this group of genes nevertheless might still qualify for a DNB since they matched two out of the three criteria used to identify DNBs. Their I value first sharply increased before returning to lower values. This rise is mostly due to a sharp decrease in PCCout between 0 and 8 h, accompanied by a more modest increase in CV. As mentioned, the internal correlation value PCCin decreased, and therefore was not driving the I value. One must note that we computed a Pearson correlation coefficient as advocated [51]. We also tried a Spearman correlation value, which showed a slightly different behavior with a modest increase in PCCin between 8 and 24 h and continued to increase steadily up to 72 h, not affecting the global surge in I value (not shown).

### The Initial Driver Genes belong to the Sterol Synthesis Pathway

Since we observed major changes after 8 h of differentiation, one asked how early changes in gene expression could be detected. For this we performed a second single-cell kinetic experiment, where we obtained the expression level of 90 genes in 48, 48, 39, and 41 single cells from 0, 2, 4, and 8 h of differentiation, respectively.

**Fig 7:**
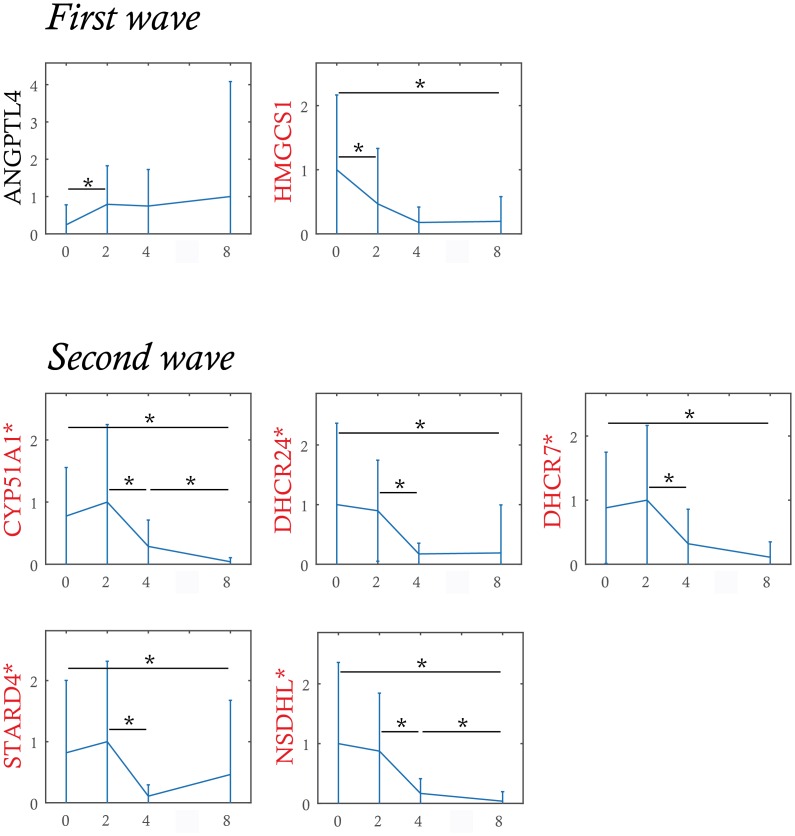
Initial expression waves analysis. Genes are sorted according to the time of the first significant expression variation. The first wave corresponds to genes with a significant variation detected during 0 h and 2 h. The second wave corresponds to genes with a significant variation detected during 2 h and 4 h but without significant variation detected earlier. Genes labeled in red belong to the group of genes associated with sterol synthesis. Significant variations (-*-) are detected by non-parametric Mann-Whitney test (*p*-value < 0.05) if the test is positive in more than 90% of 1,000 bootstrap samples. Genes prefixed by * have a significant variation between 0 h and 8 h detected in both experiments (0 to 72 h, as well as 0 to 8 h). The probability of having 6 genes over 7 (in the first and second waves) belonging to the 10 sterol cluster genes among all 90 genes is estimated to *p* = 1.8 × 10^−6^ with the hypergeometric probability density function. Data for this figure can be found at osf.io/k2q5b.

We then defined the first wave of response as genes that showed a significant difference between 0 and 2 h. Two genes satisfied this criterion (Fig 7), establishing that the transcriptional response to the medium change was a very fast process, but concerned only a very limited number of genes.

The second wave was defined as genes not belonging to wave 1 and showing a significant difference between 2 and 4 h of the response. Five genes satisfied this criterion (Fig 7). It was remarkable that six out of the seven genes from waves 1 and 2 belonged to the same functional group, that is the group of genes associated with sterol synthesis. This proved to be highly statistically significant (*p* = 1.8 × 10^−6^). We therefore can propose that the sterol synthesis pathway could act as one of the drivers of the changes that will update the internal network from the changes in external conditions. This would be in line with our previous demonstration for the role of cholesterol synthesis in the decision making process in our cells [35].

### A Surge in Cell-to-Cell Variability

**Fig 8:**
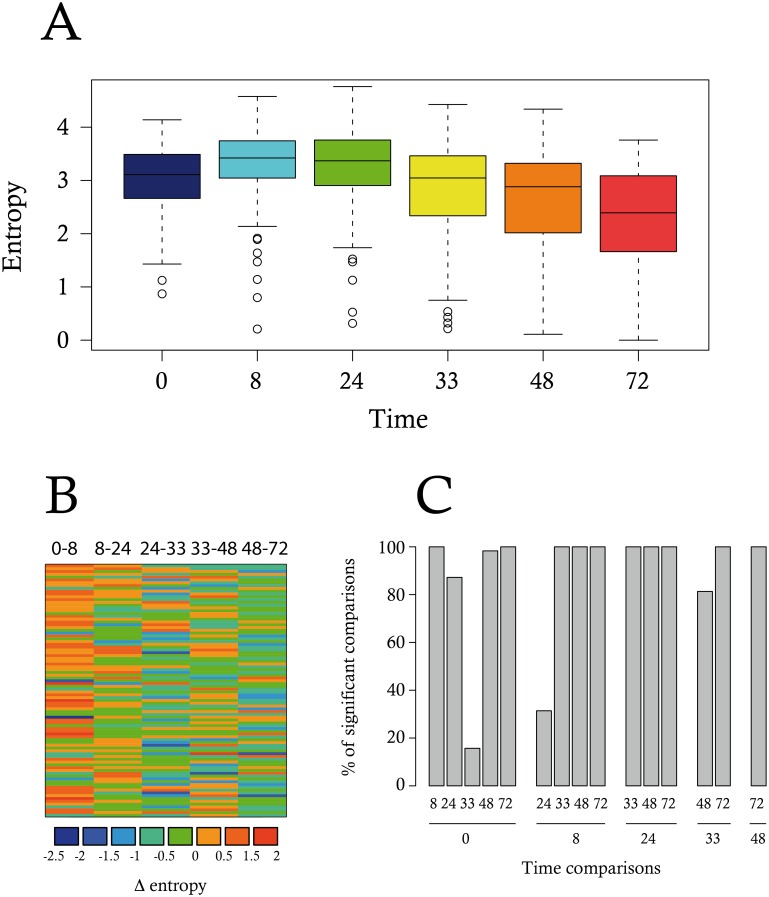
Cell-to-cell heterogeneity measurement using Shannon entropy. (A) A Shannon entropy was calculated for each time-point for each gene. Boxplots represent the distribution of the entropy values; (B) Gene entropy variation: for each gene (i.e., lines), we represented the difference between entropy values at two consecutive time-points (Δ-entropy) using a color gradient code. Negative and null delta entropies (i.e., for a given time-point, the entropy value for these genes decreased or does not change, compared to the earlier time-point) are colored in blue and green. Positive delta entropies are colored in orange or red; (C) We assessed the significance of the differences between any pair of time-point through a Wilcoxon test. The robustness of the result was assessed by performing subsampling. The barplot shows the results as the percentage of 1,000 iterations for which a significant difference (*p*-value < 0.05) was detected. Data for this figure can be found at osf.io/k2q5b.

A critical novel opportunity provided by single-cell analysis is to study cell-to-cell variability of gene expression as an observable per se and also to add new insight to characterize the temporal progression of differentiation. The question as to what may be the best metrics for quantifying gene expression variability is still open. An aggregated measure called the Jensen-Shannon divergence has been proposed previously as a measure for gene expression noise [9]. One of the main drawbacks of this metric is that it was not possible to assess whether or not the differences observed were statistically significant. We therefore decided to use a simpler Shannon measure of the heterogeneity among the cells for their gene expression profile (see Materials and Methods and S2 Fig). Such a measure provided a distribution of entropy values per gene per time-point, allowing to perform statistical tests. We observed that this entropy increased gradually along the differentiation process, reaching its maximal value at 8 to 24 h, before declining toward 72 h (Fig 8A).

Such an increase of entropy between 0 and 8h resulted from a global increase of each gene entropy, except for a few (Fig 8B). The observed rise in entropy value was highly significant as early as 8 h when compared to 0 h of differentiation. Furthermore, decrease in entropy also became significant between 24 and 33 h of differentiation (Fig 8C). Consequently, since entropy can be defined as a measure of the disorder of a system, this result suggested that a maximal heterogeneity was achieved at 8–24 h of the differentiation process in the expression of our 90 genes, before significantly decreasing to a much lower level of heterogeneity.

### Potential Explanation for the Rise in Variability

Different potential causes can be envisioned to explain this increase in entropy, including cell size and cell-cycle stage variations, asynchrony in the differentiation process, and more dynamical causes.

**Fig 9:**
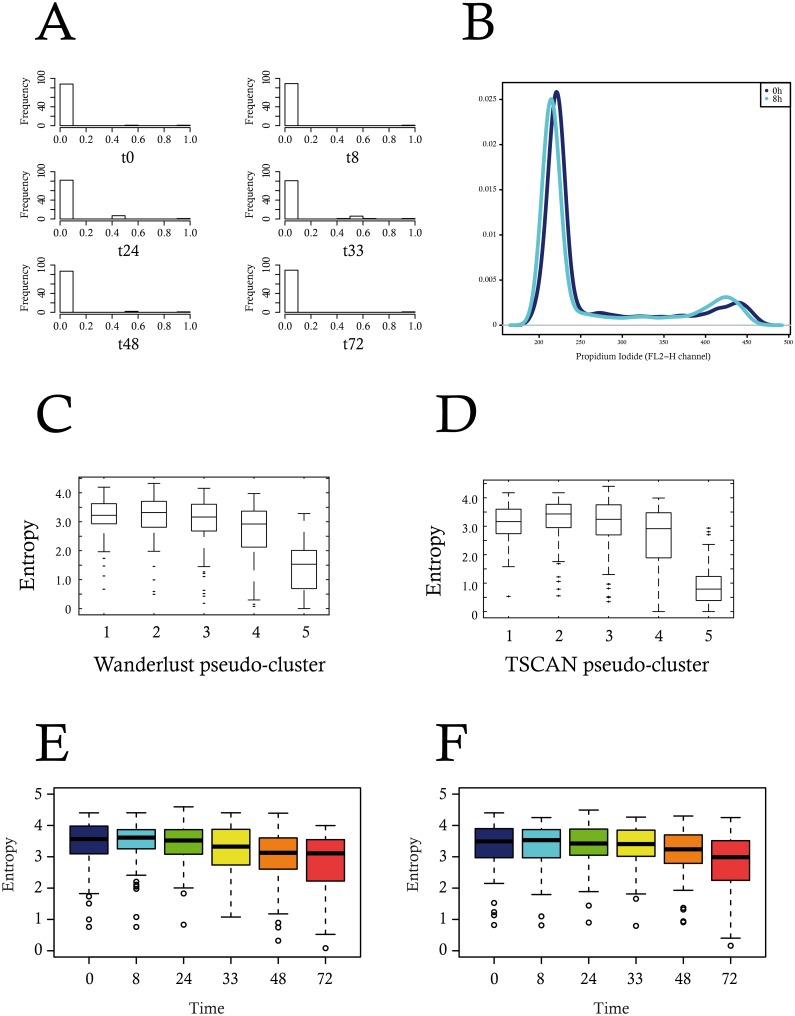
Exploration of potential cofounding factors. (A) Correlation of the CTCF gene with the rest of the 91 genes, at all six time-points. (B) FACS analysis of the cell cycle repartition at 0 and 8 h of differentiation. The difference between the two distributions was found not to be statistically significant (*p* = 0.18 using a Wilcoxon test). (C and D): calculation of the entropy content per cluster of cells re-organized using either WANDERLUST (C) or TSCAN algorithm (D). (E and F) In silico comparison of the effect of a synchronous versus an asynchronous differentiation process on the evolution of entropy. Data for this figure (C to F) can be found at osf.io/k2q5b.

**Fig 10:**
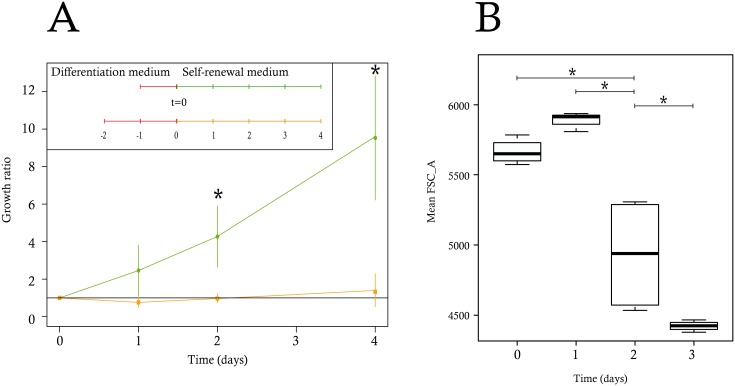
Evolution of physiological differentiation parameters. (A) T2EC were induced to differentiate for 24 and 48 h and subsequently seeded back in self-renewal conditions. Cells were then counted every day for 5 d. The green curve represents the growth of cells induced to differentiate for 24 h and the orange curve indicates the growth of cells induced to differentiate for 48 h. The data shown are the mean +/− standard deviation calculated on the basis of three independent experiments for the time-points 72 h and 96 h and four experiments for all other time-points. The growth ratio was computed as the cell number divided by the total cells at day 0. The significance of the difference between growth ratios at 24 h and 48 h was calculated using a Wilcoxon test. (B) The boxplots of the mean size observed were based on four independent experiments, each using 50,000 cells, using FSC_A as a proxy for cell size. All of the variances were compared by pairs using the F test and the * indicates when the variances were significantly different. Data for this figure can be found at osf.io/k2q5b.

As suggested in some previous works, cell size and cell-cycle stage variations could influence gene expression, and become confounding factors [67–69]. Nevertheless, variability due to variations in cell cycle has been shown to be quantitatively negligible in erythroid precursors [70]. We also added in our gene list the CTCF gene, known to be cell-cycle regulated in chicken cells [71]. Almost no correlation was detected between this gene and any of the 91 other genes (Fig 9A) demonstrating that our gene list contained virtually no other cell-cyle-regulated gene. Furthermore, we assessed whether or not the repartition of our cells within the different phases of the cell cycle could have been modified at a time where entropy was peaking. No significant difference in cell cycle repartition could be seen at 8 h of differentiation (Fig 9B). Altogether, those results demonstrate that a potential effect of cell cycle variation would only marginally explain our data. Regarding cell size, it is important to note that in our system the peak in gene expression variability at 8–24 h occurs at a time where cell size is not affected (Fig 10B). If anything, we observed a slight increase in cell size, which could be responsible for a decrease, and not an increase, in noise [72].

We then assessed a potential effect of asynchrony in the differentiation process. For this, we first employed the following algorithms: SCUBA [52], WANDERLUST [53] and TSCAN [54] to reorder the cells according to the calculated pseudotimes. However, SCUBA led to a cell re-ordering that was highly inconsistent with the actual time-points, where all self-renewing cells (time 0 h) were placed in the middle of the SCUBA order (not shown). WANDERLUST and TSCAN produced a more reasonable cell ordering. However, the trajectories of the gene expression profiles following this ordering were quite erratic (not shown). Nevertheless, the entropy of sub-populations of cells, grouped according to either their WANDERLUST pseudotimes or TSCAN clusters, showed the same rise-then-fall profile as with the original single cell data (Fig 9C and 9D).

In theory, these algorithms are supposed to reconstruct a posteriori the “hidden” order along the differentiation pathway. Within this frame, the behavior of entropy in re-ordered cells tends to support the idea that asynchrony in the differentiation process is not the leading cause of our observed increase in entropy.

However the intrinsic burstiness of the gene expression process [24, 73–75] might cause some issues in the use of cell re-ordering algorithms. We therefore examined this question by using a more formal approach. We reasoned that a modeling strategy might be useful in establishing the role asynchrony might play, especially since forcing a synchronous differentiation is not accessible in vitro, but can be done in silico. We used a two-state model of gene expression [27, 39–41, 56], for which we could learn the parameters from the data (see Materials and Methods). In the synchronous case, we obtained a variation in entropy resembling the one we calculated from the data (Fig 9E). The introduction of asynchrony induced a flatter time profile of the entropy (Fig 9F).

This finding did not, however, prove that our cells are synchronously differentiating, but only demonstrated the effect of asynchrony: in the background of bursty gene transcriptional process, asynchrony will tend to smoothen (and not augment) the entropy of the system. Therefore the observed surge in entropy can not be attributed to the asynchrony of the process.

The rise-and-fall of entropy in our data is in line was examined in a different setting, namely a reprogramming process [58]. The authors stated, “The initial transcriptional response is relatively homogeneous,” offering the opportunity to examine the entropy time profile in such a homogeneous process. Our analysis of this dataset produced a similar behavior for entropy which significantly increased initially, before returning to lower values (S8 Fig).

Altogether our analysis is compatible with the notion that the rise and fall in entropy is the consequence of the dynamical behavior of the underlying gene regulatory network.

### The Point of No Return in T2EC Differentiation is Located between 24 h and 48 h

The above analysis of single-cell transcript profiles displays the following pattern:

1. A decrease in correlation value is observed between 0 and 8 h, and then correlation increases between 24 and 72 h.
2. An increase in I score value is observed between 0 and 8 h, then a return to its initial value at about 33 h, before continuing to decrease gradually.
3. A surge in entropy is significant at 8–24 h, and significantly decreases between 24 and 72 h.

Altogether, those results point toward the 8 and 24 h time-points as being a possible decision point, hence, a “point-of-no-return” in the differentiation process, beyond which cells are irreversibly committed toward erythrocytic differentiation. Consequently, we hypothesized that committed cells would be unable to revert back to a self-renewal process after 24 h of differentiation. To test this hypothesis we induced T2EC to differentiate for 24 h or 48 h, after which cells were transferred back into the self-renewal medium, in order to determine whether or not cells could revert back to the undifferentiated state after they had received differentiation signals for a given period of time. We observed that T2EC induced to differentiate for 24 h were still able to self-renew upon change of medium, while cells induced for 48 h could not do so (Fig 10A).

T2EC induced for 48 h seemed to stay in a quiescent state until they died. We therefore concluded that the physiological point of no return is located between 24 h and 48 h of our differentiation process, as suggested by our in silico analysis. Finally we determined whether cell size, a phenotypic integrated variable that has historically been used to monitor erythroid maturation [76, 77] would manifest the behavior of the underlying molecular network with respect to cell-cell variability. We therefore assessed cell size variation during the differentiation process. As expected [32], mean cell size started to decrease during differentiation to reach a minimum by 72 h (Fig 9B). Interestingly, cell size variability significantly peaked at 48 h before dropping precipitously by 72 h. Thus the high variability of gene expression observed at 24 h preceded a significant peak in cell size variability 1 d later.

## Discussion

In the present work we assessed, using single-cell RT-qPCR, the temporal changes of gene expression in individual cells in a population of cells undergoing differentiation. For this, we used a physiologically relevant cellular system, which presents three main advantages: (i) those cells are primary, non-transformed cells; (ii) they do not show any tendency to spontaneous differentiation; and (iii) they can only differentiate along the erythrocytic lineage, excluding heterogeneity arising from coexistence of cells differentiating along different lineages.

To quantitatively assess the role of gene expression variability, we first defined a subset of genes relevant for analyzing the differentiation process. At the level of whole-population analysis this gene subset allowed a clear distinction among differentiation time-points. However, when assessed at the single-cell level, our analyses revealed a much higher cellular heterogeneity. Despite this heterogeneity, the selected genes were still effective in separating the two most extreme time-points in T2EC differentiation, confirming that information associated with the differentiation process is embodied in the gene expression data at the single-cell level. From the dataset that we generated at the single-cell level, two main results could be obtained: (i) regarding the biology of the erythroid differentiation, we identified previously unidentified genes as being important components of the self-renewal and differentiation of erythroid progenitors, and (ii) on a larger perspective, our results fully supported a dynamical view where differentiation can be seen as a critical phase transition driven by stochasticity.

### Identification of new genes involved in the erythroid differentiation process

One question deals with the possible identification of important genes that can be seen as “drivers” of the process. At least three list of genes were generated during the course of this work that may qualify:

1. the “early drivers,” genes identified in the wave analysis;
2. the genes qualifying for the DNB, and
3. the most densely connected genes in the correlation graph;

Restricting only to the most densely correlated genes at 0 and 8 h (since the two other lists were validated on those time-points), one observed a partial overlap between the three lists (S9 Fig), with no gene being common to all three lists. One possible explanation is simply that the three lists were obtained through different approaches, not supposed to identify the same set of genes. This result nevertheless suggests that although all of those genes might be functionally important for the differentiation process, they might be involved in the global response at different levels. The early drivers might be more important for informing the whole network at early time points, whereas the two other genes sets might be involved in a more global reconfiguration of the network at later time-points. In any case those gene lists are to be seen as traces resulting from the behavior of the underlying dynamical network, and should not be mistaken for the dynamical network itself. It would therefore be of utmost importance to be able to correctly infer such a network. We are actively pursuing this goal in our group.

We discuss below possible functions of some of those genes, a full discussion for all genes being out of the scope of the present paper.

As previously mentioned, *Sca2* is a gene which we have previously shown to be associated with the self-renewal of erythroid progenitors [34].

*LDHA* encodes an enzyme that catalyzes the conversion of pyruvate to lactate, and has been involved in the Warburg effect (or anaerobic glycolysis), which is the propensity of cancer cells to take up glucose avidly and convert it to lactate [78]. Furthermore, deletion of *LDHA* has been shown to significantly inhibit the function of both hematopoietic stem and progenitor cells during murine hematopoiesis [79].

Since *LDHA* expression is under the control of HIF1*α* transcription factor [79], it could be involved in the response of immature erythroid progenitors to anemia. Those cells have to show a significant amount of self-renewal for recovering from a strong anemia, implying low oxygen condition [80]. It makes perfect sense that in this case the metabolism of self-renewing progenitors would rely upon an anaerobic pathway.

Moreover, HIF1alpha has also been shown to be an upstream regulator of HSP90*alpha* secretion in cancer cells in a protective way against the hypoxic tumoral environment [81]. Therefore, our results are in line with other findings showing that anaerobic glycolysis is favored in hypoxic conditions, such as the bone marrow environment, and required for stem cell maintenance [82]. Otherwise, since *LDHA* and *HSP90alpha* form part of the lists of potentially important genes between 0 and 8 h, our finding suggests that erythroid differentiation might be accompanied by a change from anaerobic glycolysis toward mitochondrial oxidative phosphorylation, as recently proposed [83].

Finally, our analysis highlighted the importance of the sterol synthesis pathway in the self renewal process since:

1. Among genes identified by RNAseq whose expression changed significantly, we found different genes associated to the sterol synthesis, such as *HMGCS1*, *CYP51A1*, *DHCR24*, *DHCR7*, *STARD4*, and *NSDHL* (S4 Fig);
2. The expression of those genes decreased promptly after the change of the external conditions, i.e the induction of the differentiation (Fig 7);
3. *STARD4* was both an early driver and one of the genes that displayed the highest number of edges at 0 h (Fig 5C). It has recently been demonstrated that *STARD4* expression could be used as poor prognosis gene in a six genes signature that defines aggressive subtypes in adult acute lymphoblastic leukemia [84].

These observations support the importance of sterol synthesis in the maintenance of cellular self renewal state and the necessity of a decrease of some sterol associated genes expression to allow the differentiation. The question as to why this group of genes act as the early sensors of change in environmental conditions remains elusive. In line with our previous results [35], one could hypothesize that cholesterol synthesis is a barrier toward differentiation/apoptosis that has to be lowered for differentiation to proceed.

### A functional role for the surge in gene expression during critical transition?

On a more global perspective, the importance of cell-to-cell heterogeneity as a “biological observable” at the single-cell level, even among cells classified as belonging to the same “cell type” [85], is increasingly recognized [86]. But to what extent and when is such heterogeneity functionally important? Most single-cell transcript profile analyses of cell populations have so far focused mostly on computational descriptive analysis to identify clusters, and temporal progression, or to test dimensionality reduction and visualization tools, but less so to test a biological hypothesis. Here we used the single-cell granularity of gene expression analysis to test the long-standing hypothesis that stochastic cell-cell variability is not simply the byproduct of molecular noise but that such randomness of cell state plays a key role in differentiation [28]. In this Darwinian view, differentiation starts with an unstable gene expression pattern, generating cell type diversity. Therefore, one testable prediction was that an increase in gene expression heterogeneity should be observed during the critical phase of cell differentiation whenever the irreversible decision to commit is made.

Our main contribution is a demonstration that the increase in molecular variability precedes critical functional variations in cellular parameters, most importantly including the commitment status of the cells. Taken together, the timing of three observables achieved at single-cell resolution provides a coherent picture of a temporal structure of differentiation that would be invisible to traditional whole-population averaging techniques: (i) the surge in cell-to-cell variability of gene expression patterns of individual cells at 8–24 h; (ii) a sudden drop in the overall correlation, concomitant with the emergence of a DNB; and (iii) followed by the phenotypic marker of differentiation, the decrease of cell size, for which variability peaks at 48 h.

An important question is the relevance of that peak in variability. We demonstrated experimentally that no cell was able to return to a self-renewal state after 48 h in a differentiation medium. A similar timing for point-of-no return has previously been suggested in FDPC-mix cells [87]. Such an irreversible commitment to differentiation preceded by a highly significant increase in cell-to-cell variability is consistent with the explanation that cells differentiate by passing through two phases [87]: a first phase in which the self-renewing state is destabilized and primed by perturbation of their extracellular environment, followed by a second phase of a stochastic commitment to differentiation.

These observables (emergence of a DNB, drop in correlation, significant increase in entropy, surge in cellular parameters variations) jointly suggest a critical state transition, a class of dynamical behaviors that has been proposed to explain the qualitative, almost discrete and noise-driven “switching” into a new cell state as embodied by differentiation [88]. This conceptual framework naturally explains the irreversibility of fate commitment [89]. Indeed the maximum of the above three observables coincided with the functionally demonstrated point-of-no return to the self-renewal state in T2EC differentiation process, which was located between 24 and 48 h.

From a more biological perspective, we can view differentiation induction as a process of adaptation in which the cell’s internal molecular network, adapted for growth in self-renewal conditions, has to adjust to the new external conditions when differentiation is induced by the change in external conditions. For example, in yeast, it has been shown that a nonspecific transcriptional response reflecting the natural plasticity of the regulatory network supports adaptation of cells to novel challenges [90]. The underlying mechanisms are yet to be discovered, but one would expect global mechanisms to be involved. Modifications of the chromatin dynamics [27] under the possible control of metabolic changes [91] are obvious candidates for such a role. Fluctuation in important transcription factor level has also been proposed to be involved [92]. The surge of non-specific variability would allow exploration of new regions in the gene expression space. Preventing such an increase in variability has been associated to trapping cells in an undifferentiated state [93]. This increase would lead to a reconfiguration of the gene expression network into a state which is compatible with differentiation conditions and which is robust and consistent with a new attractor state in the network [29]. Then the decrease of molecular variability might reflect the implementation of the fully differentiated phenotype as cells settle down in the next stable state.

In this study, we exploited the wealth of information available in single-cell data by highlighting the critical molecular changes occurring along the differentiation sequence. First, the initial gene expression waves might represent a very early signal that happens between 0 and 8 h, followed by a pre-transition warning signal revealed by the DNB analysis, concomitant with the drop in gene correlations and the rise in cell-to-cell variability. Such a pattern are thought to reflect the underlying dynamical molecular mechanisms that drives the evolution of cells through the differentiation process. The first signals could be seen as an adaptative response to environmental changes, as suggested above, whereas the last warning signal, before irreversible commitment, could be seen as the point of cell decision making. At that stage it is hard to really be sure that the DNB genes actually drives the critical transition, but at the very least they represent a clear signal that our cells are experiencing such a transition. Until 24 h, at least, cells would still be able to functionally respond to self-renewal signals. This implies that at that stage the state of the network would be compatible with both a differentiation and a self-renewal process. One of the remaining challenging questions is what makes the cell takes the irreversible decision to differentiate at a point when the system seems to be totally disorganized. We strongly believe that this will be an emerging properties from the behavior of dynamical high-dimensional molecular network.

While the current study offers a single-cell resolution view on gene expression, it does so only through snapshots at strategically selected time-points. In the future it would therefore be of great importance to obtain a continuous measurement of the underlying gene expression network in order to explain the state changes in individual cells and to reconstruct the entire trajectory of each cell in gene expression state space. This information would expose the actual process of diversification that leads to the maximal heterogeneity marking the point of no return of differentiation.

**NOTE ADDED IN PROOF:** During the submission of this manuscript we became aware of the work of Mojtahedi, et al., 2016 (doi: 10.1371/journal.pbio.2000640) which arrived at a similar conclusion, and we cite that work in our discussion.

## Materials and Methods

### Cells and Culture Conditions

T2EC were extracted from bone marrow of 19-d-old SPAFAS white leghorn chickens embryos (INRA, Tours, France). These primary cells were maintained in self-renewal in LM1 medium (*α*-MEM, 10% Foetal bovine serum (FBS), 1 mM HEPES, 100 nM *β*-mercaptoethanol, 100 U/mL penicillin and streptomycin, 5 ng/mL TGF-*α*, 1 ng/mL TGF-*β* and 1 mM dexamethasone) as previously described [32]. T2EC were induced to differentiate by removing the LM1 medium and placing cells into the DM17 medium (*α*-MEM, 10% foetal bovine serum (FBS), 1 mM Hepes, 100 nM *β*-mercaptoethanol, 100 U/mL penicillin and streptomycin, 10 ng/mL insulin and 5% anemic chicken serum (ACS)). Differentiation kinetics were obtained by collecting cells at different times after the induction in differentiation.

### Cell Population Growth Measurement

Cell population growth was evaluated by counting living cells using a Malassez cell and Trypan blue staining.

### Propidium Iodide Staining

T2EC in self-renewal medium and T2EC induced to differentiate during 8 h were incubated for 30 min on ice with 100% cold ethanol, and then 30 min at 37°C with 1 mg/mL RNase A (Invitrogen). Propidium Iodide (SIGMA) was added at 50 μg/mL 2 min prior to analysis and fluorescence was measured with the BD FacsCalibur 4-color flow cytometer, using the FL-2 channel. Data files were then extracted and analyzed using the bioconductor flowCore package.

### T2EC Collection by Flow Cytometry

T2EC were collected individually in a 96-well plate using a flow cytometer (Facs ARIA I). Each individual cell was immediately gathered into a lysis buffer (Vilo [Invitrogen], 6U SUPERase-In [Ambion], 2.5% NP40 [ThermoScientific]), containing also Arraycontrol RNA spikes (Ambion). After collection, single-cells were immediately frozen on dry ice and stored at -80°C.

### Total RNA Extraction

Cell cultures were centrifuged and washed with 1X phosphate-buffered saline (PBS). Total RNA were extracted and purified using the RNeasy Plus Mini kit (Qiagen). Then, RNA were treated with DNAse (Ambion) and quantified using the Nanodrop 2000 spectrophotometer (Thermoscientific).

### RNA-Seq Libraries Preparation

RNA-Seq libraries were prepared according to Illumina technology, using NEBNext mRNA library Prep Master Mix Set kit (New England Biolabs). Libraries were performed according to manufacturer’s protocol. mRNA were purified using NEBNext Oligo d(T)25 magnetic beads and fragmented into 200 nucleotides RNA fragments by heating at 94°C for 5 min, in the presence of RNA fragmentation Reaction Buffer. Fragmented mRNA were cleaned using RNeasy MinElute Spin Columns (Qiagen). Double strand cDNA were obtained by two-step RNA reverse transcription (RT) with random primers and purified using Magnetic Agencourt AMPure XP beads. To produce blunt ends, purified cDNA were incubated with NEBNext End Repair reaction buffer and NEBNext End Repair enzyme mix for 30 min at 20°C. cDNA were purified again using Agencourt AMPure XP beads, and dA-tail were added to these cDNA fragments by incubating them with NEBNext dA-Tailing reaction buffer and klenow fragment for 30 min at 37°C. After purification of the dA-tailed DNA, illumina adaptators were ligated to cDNA in the presence of NEBNext quick ligation reaction buffer, quick T4 DNA ligase, and USER enzyme. After size selection, purified adaptor-ligated cDNA were enriched by PCR with NEBNext High-fidelity 2X PCR Master mix, universal PCR primers and Index primers, and using thermal cycling conditions recommended by manufacturer’s procedure. Finally, enriched cDNA were purified and sequenced by the Genoscope institute (Evry, France).

### RNA-Seq Library Analysis

Sequencing files were loaded onto Galaxy (https://usegalaxy.org/). Quality was checked using FastQC. Groomed sequences were aligned on the galGal4 version of the chicken genome, using TopHat [36]. The resulting .BAM files were transformed into .SAM files using SAM Tools. The gene counts table was generated using HTSeq [37] and the chr_M_Gallus_gallus.Galgal4.72.gtf annotated genome version. Differential gene expression was computed using EdgeR and plotted with the plotSmear function [38].

### High-Throughput Microfluidic-based RT-qPCR

Every experiment related to high-throughput microfluidic-based RT-qPCR was performed according to Fluidigm’s protocol (PN 68000088 K1, p.157–172) and recommendations.

#### Reverse transcription of isolated bulk-cell RNA and single-cell RNA

- Isolated bulk-cell RNA
  Fifty nanograms of extracted bulk-cell RNA were reverse-transcribed using the Superscript III First-Strand Synthesis SuperMix for qRT-PCR kit (Invitrogen). The reverse transcription step and RNAse H treatments were performed according to manufacturer’s instructions. Reverse transcription was performed during 30 min at 50°C, followed by 5 min at 80°C, and RNAse H treatment was run at 37°C during 20 min. Finally, cDNA were stored at -20°C.
- Single-cell RNA
  Single-cell lysates were thawed on ice and denatured for 1.5 min at 65°C. RNA were reverse-transcribed in presence of SuperScript III Reverse Transcriptase enzyme, from the SuperScript VILO cDNA Synthesis kit (Invitrogen), and T4 gene 32 protein (New England Biolabs) to improve reverse transcription efficiency. The reaction thermal cycling conditions were 5 min at 25°C, 30 min at 50°C, 25 min at 55°C, 5 min at 60°C and 10 min at 70°C.

#### Specific target amplification of cDNA

Primers were designed using the Ensembl database (http://www.ensembl.org/Gallus_Gallus/Info/Index/) and Primer3Plus software (http://www.bioinformatics.nl/primer3plus/). For information about the primers sequences used, please contact the authors.

The cDNA pre-amplification was performed using the TaqMan PreAmpMaster (Applied Biosystems) mixed with all primer pairs of the genes of interest (Sigma-Aldrich), diluted at 500 M. For single-cell cDNA pre-amplification, this reaction mix was also composed of 0.5 M pH8 EDTA. The thermal cycling program used for single-cell cDNA is 10 min of enzyme activation at 95°C, followed by 22 cycles at 96°C for 5 s and 60°C for 4 min. For bulk-cell cDNA, the enzyme activation step was followed by 14 cycles at 95°C for 15 s and 60°C for 4 min.

#### Exonuclease treatment

Exonuclease I (*E. coli*, New England BioLabs) was used on pre-amplified cDNA to eliminate single-strand DNA. The treatment was performed at 37°C during 30 min and then the enzyme was inactivated at 80°C during 15 min. For bulk-cell, cDNA were diluted in TE (10 mM pH8 Tris, 1 mM EDTA). For single-cell, cDNA were diluted in low EDTA TE buffer (10 mM pH8 Tris, 100 nM EDTA). All samples were then stored at -20°C.

#### RT-qPCR: data generation

Pre-amplified cDNA were mixed with Sso Fast EvaGreen Supermix With Low ROX (Bio-Rad) and DNA binding dye sample loading reagent (Fluidigm). Primer pairs of the genes of interest were diluted at 5 μM with the Assay Loading Reagent (Fluidigm) and low EDTA buffer. First, the 96.96 DynamicArray IFC chip (Fluidigm) was primed. Then, prepared cDNA and primer pairs were loaded in the inlets of this device.

To avoid chip-linked variability, when analyzing single-cell data we were careful to represent every time-point in each of the four microfluidic-based chip analyzed.

The prime step and transfer of cDNA samples and primers from the inlets into the chip were performed using the IFC Controller HX (BioMark HD system). The chip was analyzed using the BioMark HD reader according to the GE 96 × 96 PCR + Melt v2.pcl program, thanks to the data collection software. Then, raw data were analyzed with the Fluidigm Real-Time PCR Analysis software.

Positive exogenous controls (RNA spikes) were used to validate the RT-qPCR experiment as recommended by Fluidigm Company. We also used the RNA spikes to normalize the data (see below). To determine qPCR efficiency of every primer pairs used, serial dilution scales of bulk-cell cDNA were performed. PCR efficiencies were calculated as follows: *E* = 10^−1/slope^. Primer pairs presenting PCR efficiency less than 80% or more than 120% were removed from subsequent analyses.

#### RT-qPCR: low-level data analysis

First, a manual examination was performed regarding data quality. RTqPCR data were exported from the BioMark HD data collection software. On every microfluidic-based chip, each gene was controlled in a qualitative manner in order to keep only reliable and good quality data. For this we manually edited the data files by adding a new column named “DELETED.” Numbers “0” or “1” were appended in this column according to various criteria. Quality control was based both upon amplification and melting curves examination. For one given gene all the melting curves had to be centered on a unique melting temperature. When a given melting curve peak shifted to a higher or lower Tm, “1” was added into the DELETED column for this amplification. Moreover, data displaying a double peak were also considered unreliable and annotated with a “1.” Finally, “noisy” amplification curves departing from the smooth classical sigmoidal shape were also tagged as “1.” We allowed the quantification cycle (Cq) to be as high as 30. For a higher number of cycles, the machine returned a value of 999, meaning that there were not enough molecules to be detected. After this quality control, Cq values of data tagged as “1” were replaced with UD (for “undefined”) in the raw data file, since they would not be taken into account in later analysis. Then the new table underwent an automatic formatting consisting in a second multiple-criteria cleaning process using an in-house R script. Cq values were converted into (approximately) absolute numbers of molecules according to the following steps. First, we selected cells with at least one valid spike measurement (i.e., whose Cq is different from UD and 999). Then, we normalized the raw value ${\hat{\text{Cq}}}_{i,j}$ for cell *i* and RNA *j* according to the cell mean spike value ${\bar{\text{Cq}}}_{i}$ (or the only available spike if one is invalid), with the global mean spike value ${\bar{\text{Cq}}}_{0}$ as reference. That is, the normalized value Cq_*i*,*j*_ for cell *i* and RNA *j* is defined by

$${\text{Cq}}_{i,j}={\hat{\text{Cq}}}_{i,j}-\left({\bar{\text{Cq}}}_{i}-{\bar{\text{Cq}}}_{0}\right).$$

After removing cells with abnormally important amount of genes with low expression (high Cq_*i*,*j*_ values, suggesting the absence of a cell in the well), the numbers of mRNA molecules were estimated, considering the following: a maximum Cq equal to 30 as the measurement of 1 molecule in the well after 22 cycles of pre-amplification, a dilution factor corresponding to 1 cell extract diluted in 96 wells, and a sampling of 1/45 for PCR measurement. Thus the number m_*i*,*j*_ of RNA *j* molecules in cell *i* is given by

$${\text{m}}_{i,j}=96\times 45\times 2^{30-22-{\text{Cq}}_{i,j}}.$$

We consistently set m_*i*,*j*_ = 0 when Cq_*i*,*j*_ = 999, and m_*i*,*j*_ = UD when Cq_*i*,*j*_ = UD.

#### Replacing missing values

Since some statistical tools (like PCA) do not support missing values, the UDs had to be replaced with some *appropriate* numerical values, i.e., that do not change the data distribution, nor introduce any artificial correlation.

To this end, we calibrated the marginal distribution of each gene at each time-point using the 3-parameter Poisson-Beta family, which corresponds to the stationnary distribution of the widely-used “two-state” model of gene expression [39–41]. As emphasized in [41], it can be obtained as the mixture distribution D(a,b,c) of *X* resulting from the hierarchical model

$$\left\{\begin{array}{c} Z∼\mathrm{Beta}(a,b)\\ X∼P(cZ) \end{array}\right.$$

where *a*, *b*, and *c* are positive. Thus for each time-point *t* and each gene *j*, we estimated the parameters $a_{j}^{t}$, $b_{j}^{t}$ and $c_{j}^{t}$ by taking the absolute value of the moment-based estimators proposed in [39]. Note that these slightly modified estimators are also convergent since the parameters are assumed to be positive. This estimation was only performed for genes with at least 20 valid cells and conduced to delete genes with too many UDs. This led us to delete two genes, resulting in a total of 90 genes analysed. The data was fitted very well in practice, making it relevant to simply replace the UDs with independent samples from the corresponding distributions $D(a_{j}^{t},b_{j}^{t},c_{j}^{t})$. Considering the actual inferred parameter regime (large values of *c*, meaning that the numbers of molecules span a high range) and the continuous nature of our data, we actually ignored the Poisson step and sampled from $c_{j}^{t}\mathrm{Beta}\left(a_{j}^{t},b_{j}^{t}\right)\approx D\left(a_{j}^{t},b_{j}^{t},c_{j}^{t}\right)$.

Obviously, such artificially generated values should not be seen as data, but they ensure that the dimension-reduction algorithms perform at their best and compute relevant projection axes (e.g., the main two axes for a PCA). We checked that indeed consistent PCA outputs were generated from different UD replacement operations (not shown).

### Technical Reproducibility

Since RT-qPCR experimental procedure introduces unavoidable technical noise, we decided to explore which steps were the main sources of this variability (S1 Fig). We first assessed the reproducibility of the cDNA pre-amplification step by amplifying four cDNA samples from the same RT before analyzing it by qPCR. Gene expression levels differences between pre-amplification replicates were found to be negligible (S1A and S1B Fig). We then checked the RT-qPCR amplification step by analyzing the *RPL22L1* gene three times per chip. Expression levels between *RPL22L1* triplicates were quantitatively extremely similar (S1C to S1E Fig), confirming that amplification brings a negligible amount of variability as previously shown [42, 43]. We also tested the experimental variability induced by the RT reaction. We observed significant gene expression level differences between three RT from the same sample (S1A and S1F Fig), contrary to replicates from other critical steps. Indeed, it has been demonstrated and discussed that the RT reaction is the main source of technical noise, since it introduces biases through priming efficiency, RNA integrity and secondary structures and reverse transcriptase dynamic range [42, 44, 45]. In order to estimate the amount of variation introduced in our experiments by this step, we used external RNA spikes. The variation affecting those spikes spanned 5.8 Cqs (mean of Cq_max_−Cq_min_ across the spikes) whereas the variability affecting the genes spanned a much larger region of 22.9 Cqs (mean of Cq_max_−Cq_min_ across the genes), showing that the biological variability was much larger than the variability introduced by the RT step.

### Statistical Analysis

#### Software

Most of the statistical analyses were performed using R [46]. The *k*-means clustering was performed using the stats R library. PCAs were performed using the ade4 package [47]. All PCAs were centered (mean subtraction) and normalized (dividing by the standard deviation). All PCAs were displayed according to PC1 and PC2, which are the first and second axis of the PCA respectively. Hierarchical cluster analysis was performed applying the R hclust function, using the complete linkage method on Euclidean distances. Dendrograms were built and plotted using the dendextend R library. Correlation analysis was performed using rcorr from the Hmisc R library. The *p*-value was corrected for multiple testing using the Bonferroni method [48]. Networks were computed using Cytoscape [49]. Cross-correlation analysis was performed using the matcor function from the CCA R library. Normality of the distributions was tested using the shapiro.test function. The variances were compared using the F test with the var.test function. Wilcoxon test was performed using the wilcox.test function. t-SNE and diffusion maps were computed using the Matlab Toolbox for Dimensionality Reduction (http://lvdmaaten.github.io/drtoolbox/). The t-SNE analysis was performed on a normalized version of the data, using zscore function. Kernel PCA was computed using the Matlab kPCA script [50] applying polynomial with fractional power 0.1. All linear analysis methods (PCA, HCA and correlation analysis) were performed after applying the transformation m ↦ ln(m + 1) to the data, which gives access to the more linear Cq structure. All non-linear analysis methods (t-SNE, diffusion maps and Kernel PCA) were performed using untransformed m values.

#### I score calculation

The I score was calculated as previously described in [51] as follows: among the *n* = 90 studied genes, we defined a subset *D* containing *n*_*D*_ genes. We then defined the I score as:

$$\text{I}=\text{CV}\frac{{\text{PCC}}_{in}}{{\text{PCC}}_{out}}$$

with

$$\text{CV}=\frac{1}{n_{D}}\sum_{i\in D}{\text{CV}}_{i}\;,\;{\text{PCC}}_{in}=\frac{1}{{n_{D}}^{2}}\sum_{i,j\in D}\;C_{i,j}\;,\;{\text{PCC}}_{out}=\frac{1}{n_{D}\left(n-n_{D}\right)}\sum_{\begin{array}{c} i\in D\\ j\notin D \end{array}}C_{i,j}$$

where CV_*i*_ is the coefficient of variation of gene *i* and *C*_*i*,*j*_ stands for Pearson’s correlation coefficient between genes *i* and *j*.

#### Wave analysis

One thousand boot-strap expression matrices were generated from genes RNA counts distribution for each time-point (0, 2, 4, and 8 h). New expression matrices were generated by uniform sampling of cells, which correspond to matrix lines, using the randsample Matlab command with replacement. For each time-point combination, a Mann-Whitney U test was performed using the ranksum Matlab command to detect significant variation. Wave membership was based on time variations. By definition a gene belongs to the wave at time T if there is at least one variation detected between time T and a previous time-point and if the gene does not belong to a previous wave. Only genes identified in a wave that displayed a significant variation in more than 90% of boot-straped samples were kept in this wave.

#### Estimation of entropy

We estimated the Shannon entropy of each gene *j* at each time-point *t* as follows: we computed basic histograms of the genes with *N* = *N*_*c*_/2 bins, where *N*_*c*_ is the number of cells, which provided the probabilities $p_{j,k}^{t}$ of each class *k*. Finally, the entropies were defined by

$$E_{j}^{t}=-\sum_{k=1}^{N}p_{j,k}^{t}\log_{2}\left(p_{j,k}^{t}\right).$$

When all cells express the same amount of a given gene, this gene’s entropy will be null. On the contrary, the maximum value of entropy will result from the most variable gene expression level (S2 Fig).

#### Re-ordering algorithms

We performed the pseudotemporal ordering of cells using three different algorithms: SCUBA [52], WANDERLUST [53] and TSCAN [54]. SCUBA is a two-step cell-ordering algorithm, in which one first reduces the data dimensionality by using t-SNE [55] and then determines the principal curve in the low-dimensional projection. We applied SCUBA by reducing the data into 2-D using tSNE (perplexity = 30) and by adopting k-segments algorithm (maximal number of segments = 8) as the option for the principal curve analysis. Since the differentiation path estimated by SCUBA was undirected, we set *LDHA* as the anchor-gene/marker to define the beginning and the end of pseudotime.

In contrast, WANDERLUST is a non-branching trajectory detection algorithm [53]. The method estimates the pseudotimes by representing each single-cell as a node in an ensemble of k-nearest-neighbor graph, followed by assigning a trajectory for each graph. This trajectory is defined by connecting cells with similar gene expressions through the shortest path. To reinforce this path assembly, a set of cells is randomly chosen as waypoints. The final cell ordering corresponds to the average trajectories over the ensemble of graphs. Here, we adopted the cosine similarity distance function for the trajectory detection, in which the single cell with the maximum *LDHA* expression was used as the initial node. Each cell’s pseudotime has a value normalized between 0 and 1, reflecting its position along the differentiation path. For the entropy calculation, we grouped the cells into five pseudo-clusters, by collecting cells within five evenly spaced pseudotime window between 0 and 1 (e.g., pseudo-cluster 1 contained cells with pseudotime between 0 and 0.2, pseudo-cluster 2 contained cells with pseudotime between 0.2 and 0.4, and so on).

Finally, TSCAN is a cluster-based minimum spanning tree ordering algorithm [54]. The algorithm begins with clustering cells according to the similarity in their gene expressions, and continues with building the minimum spanning tree (MST) connecting the centroids of these clusters. The pseudotime is calculated by projecting each single cell to the MST edges. The algorithm also implements a preprocessing step involving gene clustering and dimensional reduction in order to alleviate the effect of drop-out events [54]. The preprocessing of our data produced 36 gene clusters, on which we employed the independent component analysis (ICA) to obtain a 2-D projection. Finally, we applied TSCAN using five cell clusters to generate the cell pseudotimes.

We computed the entropy for each cluster of cells following the procedure described above.

#### In silico simulations of mRNA level for single cells

In silico results were generated using the two-state model of gene expression [27, 39–41, 56]. We first inferred a set of model parameters (Kon, Koff, S0, D0) specific to each gene and depending on time. For that we used an inference method based on moment analysis [39] from our single cell expression matrix allowing to estimate three of these parameters (Kon, Koff and S0). To estimate D0 (mRNA degradation rate) we used population data of mRNA decay kinetic using actinomycin D-treated T2EC (osf.io/k2q5b). To simulate mRNA level we used the Gillespie algorithm [57]. In order to validate this modeling approach, we simulated for a given gene its mRNA evolution for 100 cells and extracted its distribution among cells at different time-points (0, 8, 24, 33, 48, and 72 h). We then compared in vitro and in silico distributions with a non-parametric Mann-Whitney U test. In silico measurements reproduced qualitatively the evolution of mean and distributions measured in vitro (not shown).

#### In silico simulations of the differentiation process

In order to stabilize the model before differentiation start, we ran the simulation for 100 h (model time) with constant parameters (value corresponding to 0 h). In silico differentiation was induced by a change in parameters values to now impose the parameters deduced from the in vitro data at different time-points. At each time step we computed parameters value with a linear interpolation between the two nearest time-points. For example at simulation time 4 h parameters values correspond to the mean value between 0 and 8 h. We simulated 100 cells at each time-point. In order to study the impact of asynchronous differentiation, we compared two situations:

1. All cells had their parameters changed simultaneously, corresponding to a synchronous differentiation.
2. We randomly chose for each cell a time lag from a uniform distribution between 0 and 24 h. Then during the simulation, parameters started to change at t = 0 h + time lag. This corresponded to an asynchronous differentiation.

We then used the same metrics for analyzing those in silico distributions as those used for analyzing the in vitro data.

#### scRNA-seq data analysis

Counting table from [58] was downloaded from the following URL: http://www.ncbi.nlm.nih.gov/geo/download/?acc=GSE67310. The original (Log2[FKPM]) data were transformed into FKPM data for analysis using the BPglm algorithm [59]. Running the algorithm with an FDR value of less than 0.00005 and using the Bonferroni correction method for multiple testing led us to a list of 776 differentially expressed genes, on which entropy was computed. Statistical significance was computed using the Wilcoxon non parametric test.

## Supporting Information

S1 Fig Reproducibility of the pre-amplification and RT-qPCR amplification steps. (A) the protocol used for assessing variation sources; (B) variations induced by four independent pre-amplifications when assessing the level of expression of the OSC gene; (C–E) variations induced by the PCR amplification step. The *RPL22L1* gene expression was analyzed three times per single-cell. Shown is the correlation between those three RT-qPCR replicates. The corresponding correlation coefficients are plotted on the graphs. The slopes of the linear regression lines are 0.99 for all three comparisons; (F) variations induced by three independent reverse-transcriptions when assessing the level of expression of the *OSC* gene.
(PDF)

S2 Fig Schematic description of the entropy value. On the left are shown gene expression values that are transformed into probabilities (pj) to observe a given expression level in a cell population. The upper case illustrates the deterministic case where all cells do express the same expression level, resulting in a probability of 1 of observing such a level. This results in a null entropy (see Materials and Methods for the calculation). The lower case illustrates the other extreme case, where all the cells have different expression level, resulting in a much higher entropy.
(PDF)

S3 Fig Scatter and MA plots showing the reproducibility of read counts between replicates and the differential expression during the differentiation process. (A,B) Relationship between biological replicates of two independent RNA-Seq experiments: self-renewing T2EC (left panel) and T2EC induced to differentiate for 48 h (right panel). For each condition, the *x*-axis represents the read counts of the first biological experiment, whereas read counts of the second biological replicate are given on the *y*-axis. Each dot corresponds to the expression level of one gene. (C) Comparative analysis of RNA-Seq data generated from two independent libraries of T2EC in self-renewing state and T2EC induced to differentiate for 48 h. The *x*-axis shows the expression level of each gene (transcript raw counts divided by the library size and multiplied by 1 million, averaged between the two independent libraries) while the fold change (self-renewal versus differentiation) appears in the *y*-axis. Red-colored dots highlights genes that are significantly differentially expressed (*p*-value < 0.05).
(PDF)

S4 Fig Identification of common patterns of expression during the differentiation process using K-means clustering. K-means clustering was used to separate the 110 selected genes into seven clusters regarding the expression profiles along the differentiation process. Starting models of gene expression pattern, corresponding to the centroid of each cluster, are represented in the first graph (starting cluster). We identified seven patterns of gene expressions with increasing, decreasing and one complex (cluster 4) dynamic profiles. The final centroid was recalculated after gene allotment, and might slightly differ from the starting one.
(PDF)

S5 Fig Representation of the 92 selected genes. (A) On the basis of RNA-Seq data and k-means analysis (S4 Fig), the 92 genes selected for the single-cell analysis (S1 Table) can be separated into three types: up-regulated (red circles), invariant (green circles), and down-regulated genes (blue circles) at 48 h of the differentiation process. For each gene (*x*-axis) the fold-change (FC) between the self-renewal state and the differentiation state at 48 h (Diff/SR) was plotted along the *y*-axis. (B) Representation of known connections among the 92 genes selected according to the STRING database (http://string.embl.de/). Each edge between two genes corresponds to a known association between those genes. The densely connected component at the center of the network graph is composed of genes involved in sterol biosynthesis. A cluster of gene encoding porteins involved in signal transduction is apparent on the top right part of the network.
(PDF)

S6 Fig Cross-correlation analysis between the gene expression value in populations and in single cells. The correlation matrix is divided into four smaller matrices: the correlation matrix of each dataset (populations: top-left panel; single-cells: bottom-right panel) and the correlation matrix between the two datasets (top-right and bottom-left panels, showing the same values). The values of the correlations are color-coded according to the scale given below. Correlation are calculated for each gene either accross populations samples or across single cells.
(PDF)

S7 Fig Distributions of the expression values for three genes up-, down-, and non-regulated during the differentiation process. The histograms show the expression distribution of three genes among single cells at 0 and 72 h differentiation time-points. The gene expression levels (m value) are shown on the *x*-axis, the number of cells (count) is represented on the *y*-axis.
(PDF)

S8 Fig Variation of entropy during a reprogramming process. We computed differential gene expression between 0 and 2 d using the scRNA-seq data from [58]. We then computed an entropy value per time-point for the 776 resulting genes. Statistical significance was computed using a Wilcoxon test.
(PDF)

S9 Fig Overlapping genes between DNBs, early drivers and correlation network nodes at 0–8 h of differentiation. The Venn diagram shows the overlap of the three lists of genes obtained from the initial expression waves analysis (green), the correlation networks (pink), and the DNB theory (blue). The common genes between these lists were searched at 0 and 8 h when all three analyses have been performed (early driver genes were only identified between 0 and 8 h).
(PDF)

S1 Table Supplementary Table 1. Shown is the complete list of the 92 genes we analyzed, together with their expression value in the four RNA-Seq libraries (SR_1 and SR_2 being the two independent libraries made using self-renewing cells and Diff_1 and Diff_2 being two independent libraries made from cells differentiated for 48 h) and the group of variation at 48 h to which they belong (up-, down-, or non-regulated).
(CSV)

## Abbreviations

CV: coefficent of variation
DNB: dynamical network biomarker
HCA: hierarchical cluster analysis
LDHA: Lactate deshydrogenase A
OSC: oxydosqualene cyclase
PC1: first principal component
PCA: principal component analysis
RT-qPCR: reverse transcription quantitative PCR
SNE: Stochastic Neighbor Embedding
