# Supplementary figures and images for: Single-Cell-Based Analysis Highlights a Surge in Cell-to-Cell Molecular Variability Preceding Irreversible Commitment in a Differentiation Process
Source: PLoS Biol. 2016 Dec 27;14(12):e1002585. doi: 10.1371/journal.pbio.1002585 (PMC5191835; doi:10.1371/journal.pbio.1002585)

A

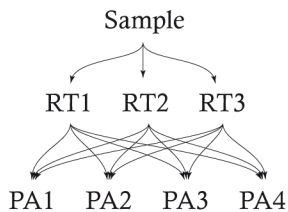

B

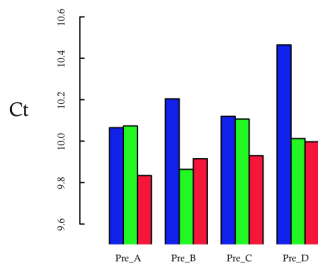

C

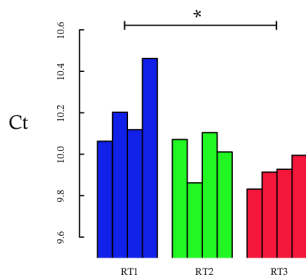

D

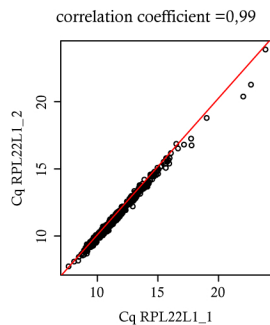

E

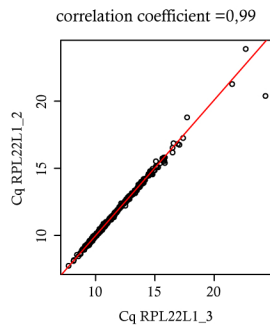

F

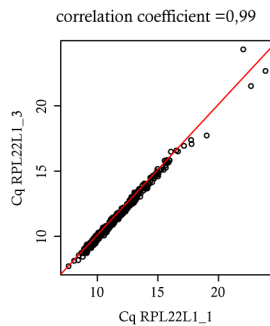

Supplement: S1 Fig — (A) the protocol used for assessing variation sources; (B) variations induced by four independent pre-amplifications when assessing the level of expression of the OSC gene; (C–E) variations induced by the PCR amplification step. The RPL22L1 gene expression was analyzed three times per single-cell. Shown is the correlation between those three RT-qPCR replicates. The corresponding correlation coefficients are plotted on the graphs. The slopes of the linear regression lines are 0.99 for all three comparisons; (F) variations induced by three independent reverse-transcriptions when assessing the level of expression of the OSC gene. (PDF) [file pbio.1002585.s001.pdf]

Expression level

Probabilities

Entropy

| Cell | m   |
|------|-----|
| 1    | 1.5 |
| 2    | 1.5 |
| 3    | 1.5 |
| 4    | 1.5 |
| 5    | 1.5 |
| 6    | 1.5 |

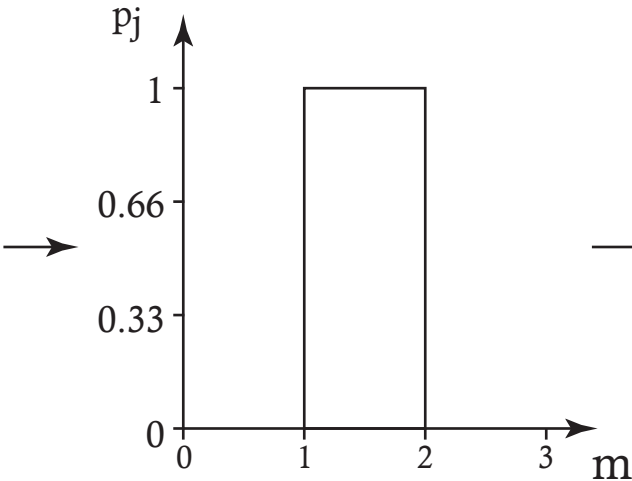

$E = 0$

| Cell | m   |
|------|-----|
| 1    | 0.7 |
| 2    | 0.3 |
| 3    | 1.5 |
| 4    | 1.8 |
| 5    | 2.1 |
| 6    | 2.4 |

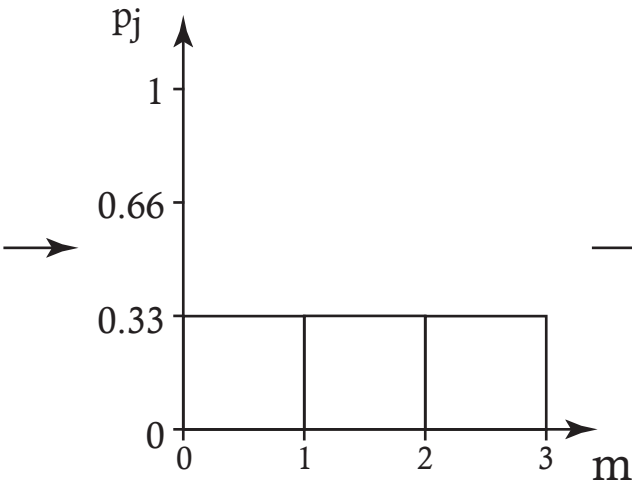

$E = 1.58$

Supplement: S2 Fig — On the left are shown gene expression values that are transformed into probabilities (pj) to observe a given expression level in a cell population. The upper case illustrates the deterministic case where all cells do express the same expression level, resulting in a probability of 1 of observing such a level. This results in a null entropy (see Materials and Methods for the calculation). The lower case illustrates the other extreme case, where all the cells have different expression level, resulting in a much higher entropy. (PDF) [file pbio.1002585.s002.pdf]

A

Self-renewal

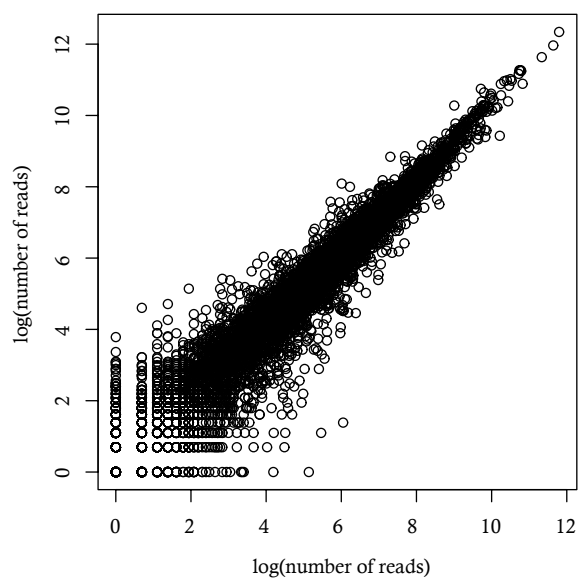

B

48 h of differentiation

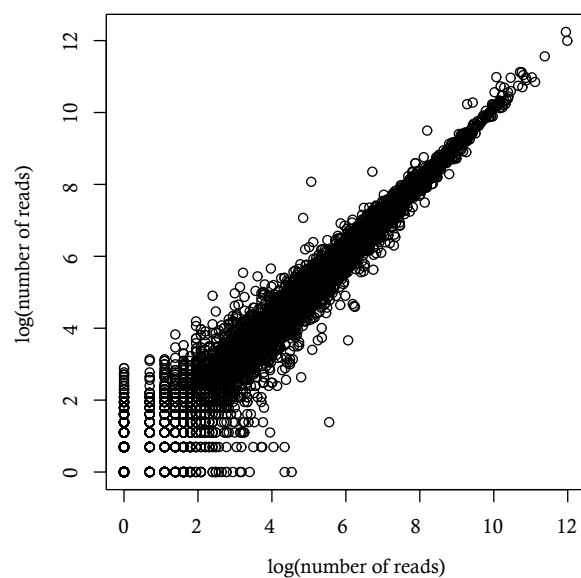

C

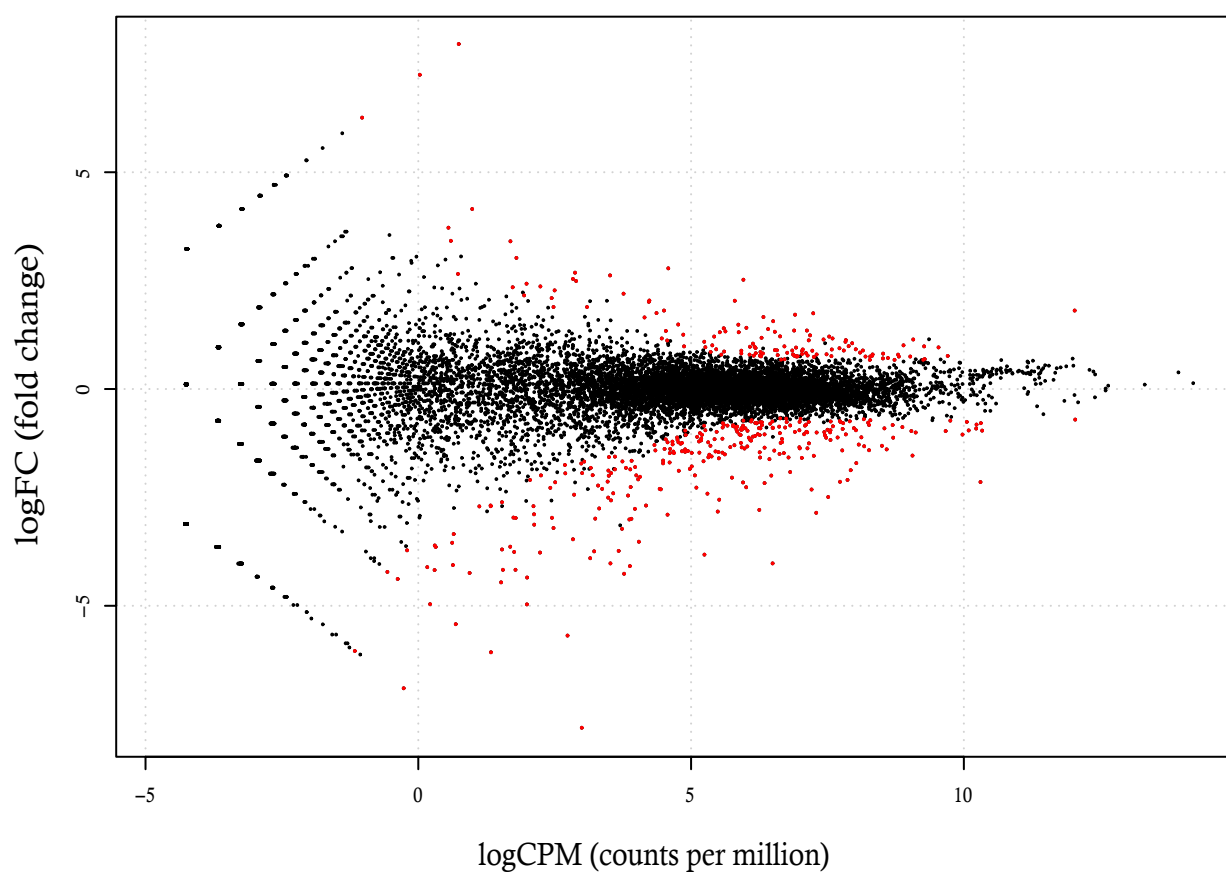

Supplement: S3 Fig — (A,B) Relationship between biological replicates of two independent RNA-Seq experiments: self-renewing T2EC (left panel) and T2EC induced to differentiate for 48 h (right panel). For each condition, the x-axis represents the read counts of the first biological experiment, whereas read counts of the second biological replicate are given on the y-axis. Each dot corresponds to the expression level of one gene. (C) Comparative analysis of RNA-Seq data generated from two independent libraries of T2EC in self-renewing state and T2EC induced to differentiate for 48 h. The x-axis shows the expression level of each gene (transcript raw counts divided by the library size and multiplied by 1 million, averaged between the two independent libraries) while the fold change (self-renewal versus differentiation) appears in the y-axis. Red-colored dots highlights genes that are significantly differentially expressed (p-value < 0.05). (PDF) [file pbio.1002585.s003.pdf]

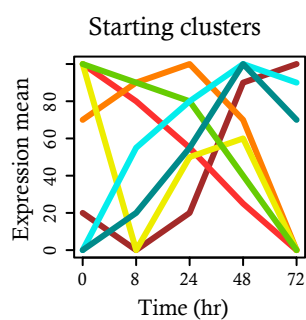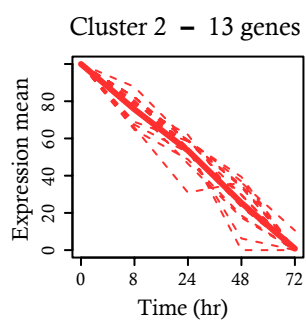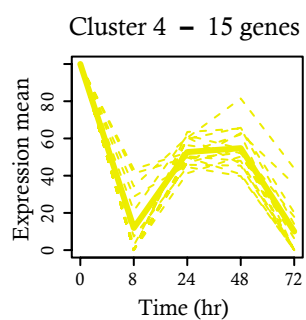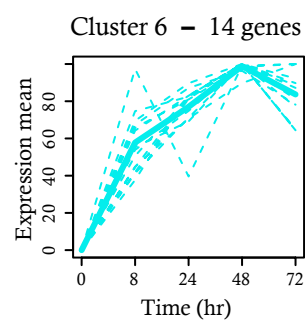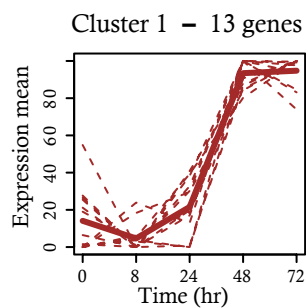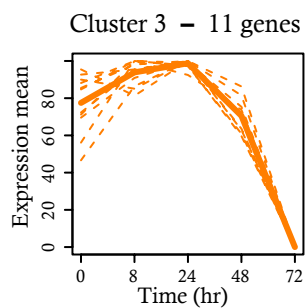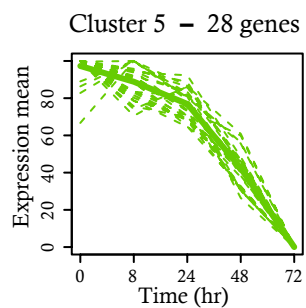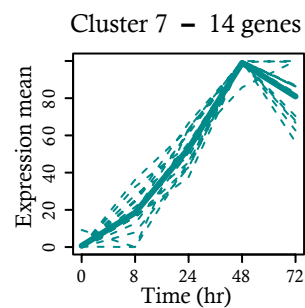

Supplement: S4 Fig — K-means clustering was used to separate the 110 selected genes into seven clusters regarding the expression profiles along the differentiation process. Starting models of gene expression pattern, corresponding to the centroid of each cluster, are represented in the first graph (starting cluster). We identified seven patterns of gene expressions with increasing, decreasing and one complex (cluster 4) dynamic profiles. The final centroid was recalculated after gene allotment, and might slightly differ from the starting one. (PDF) [file pbio.1002585.s004.pdf]

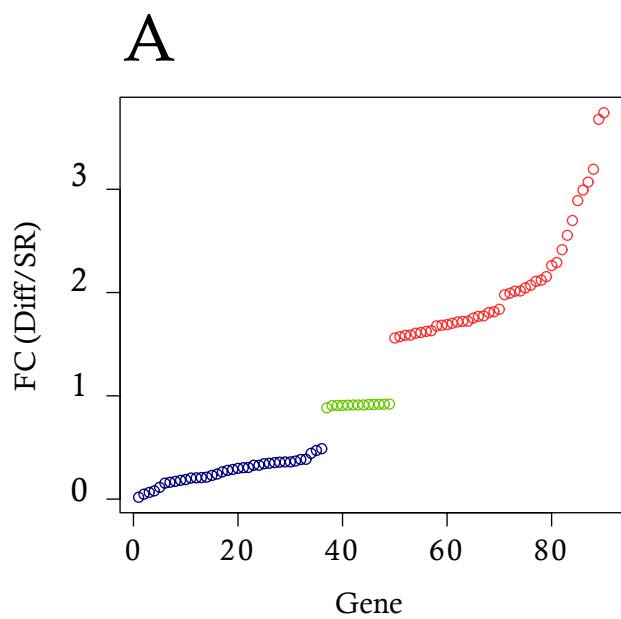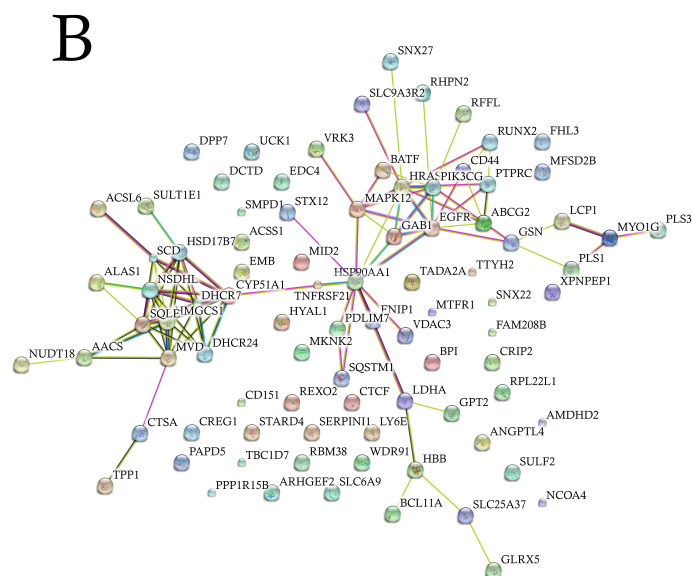

Supplement: S5 Fig — (A) On the basis of RNA-Seq data and k-means analysis (S4 Fig), the 92 genes selected for the single-cell analysis (S1 Table) can be separated into three types: up-regulated (red circles), invariant (green circles), and down-regulated genes (blue circles) at 48 h of the differentiation process. For each gene (x-axis) the fold-change (FC) between the self-renewal state and the differentiation state at 48 h (Diff/SR) was plotted along the y-axis. (B) Representation of known connections among the 92 genes selected according to the STRING database (http://string.embl.de/). Each edge between two genes corresponds to a known association between those genes. The densely connected component at the center of the network graph is composed of genes involved in sterol biosynthesis. A cluster of gene encoding porteins involved in signal transduction is apparent on the top right part of the network. (PDF) [file pbio.1002585.s005.pdf]

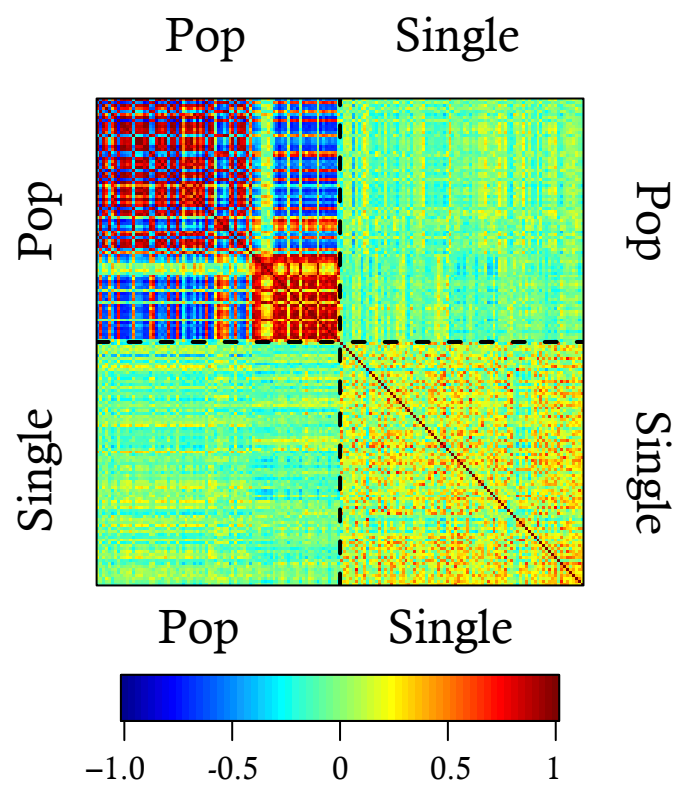

Supplement: S6 Fig — The correlation matrix is divided into four smaller matrices: the correlation matrix of each dataset (populations: top-left panel; single-cells: bottom-right panel) and the correlation matrix between the two datasets (top-right and bottom-left panels, showing the same values). The values of the correlations are color-coded according to the scale given below. Correlation are calculated for each gene either accross populations samples or across single cells. (PDF) [file pbio.1002585.s006.pdf]

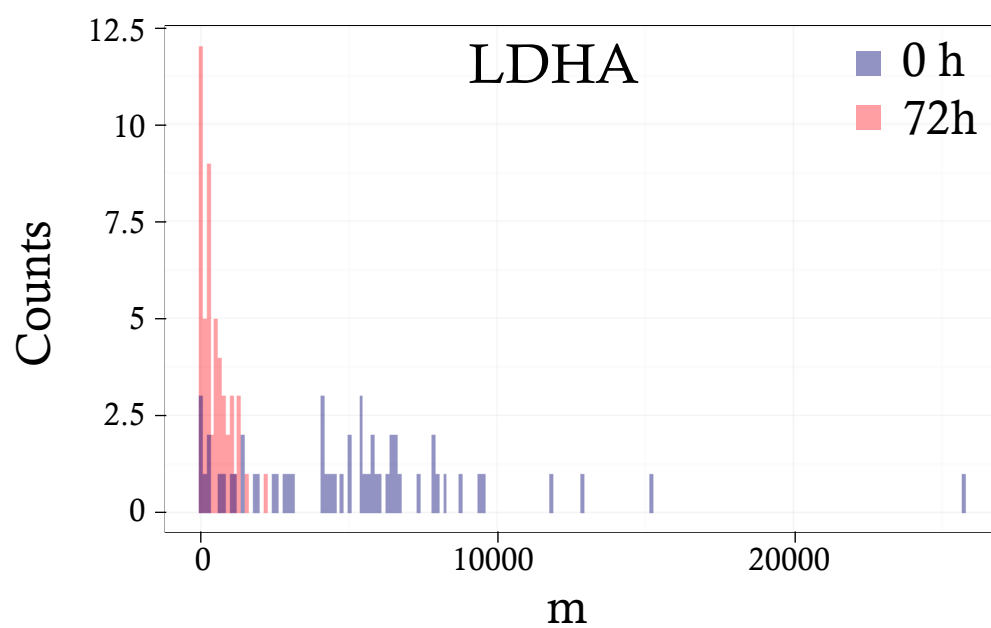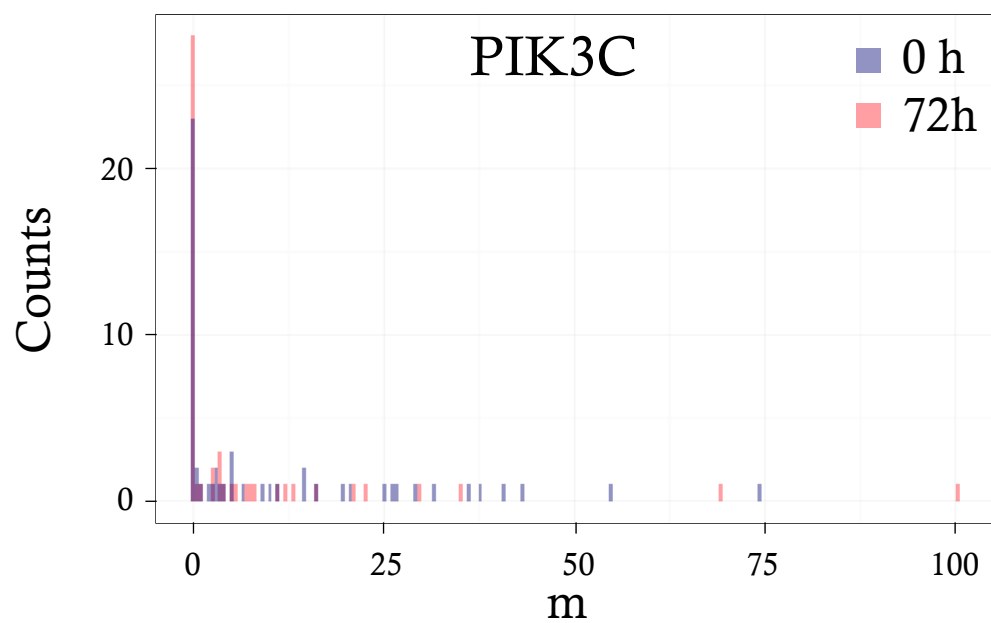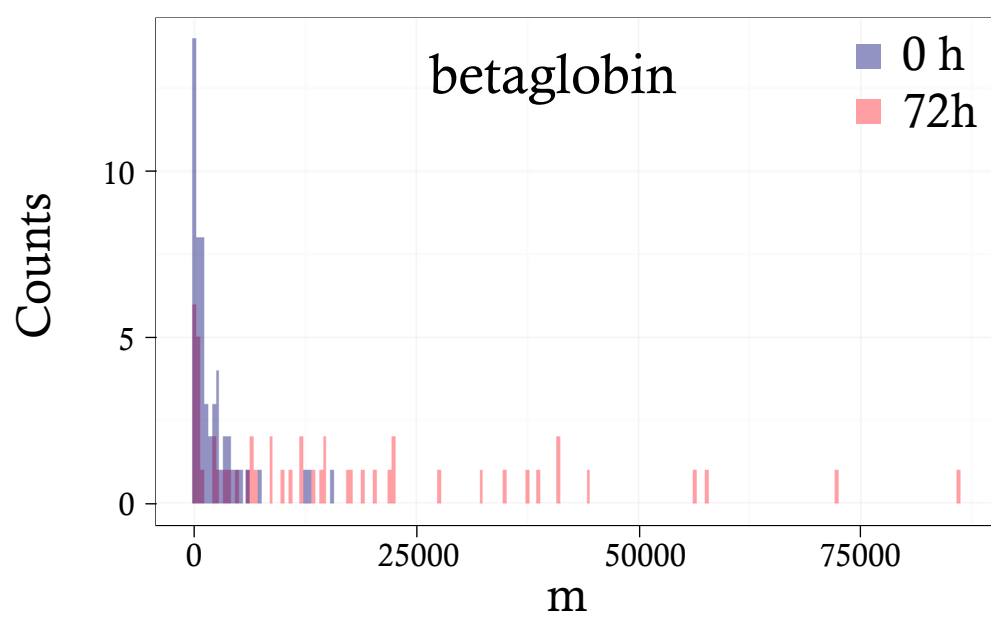

Supplement: S7 Fig — The histograms show the expression distribution of three genes among single cells at 0 and 72 h differentiation time-points. The gene expression levels (m value) are shown on the x-axis, the number of cells (count) is represented on the y-axis. (PDF) [file pbio.1002585.s007.pdf]

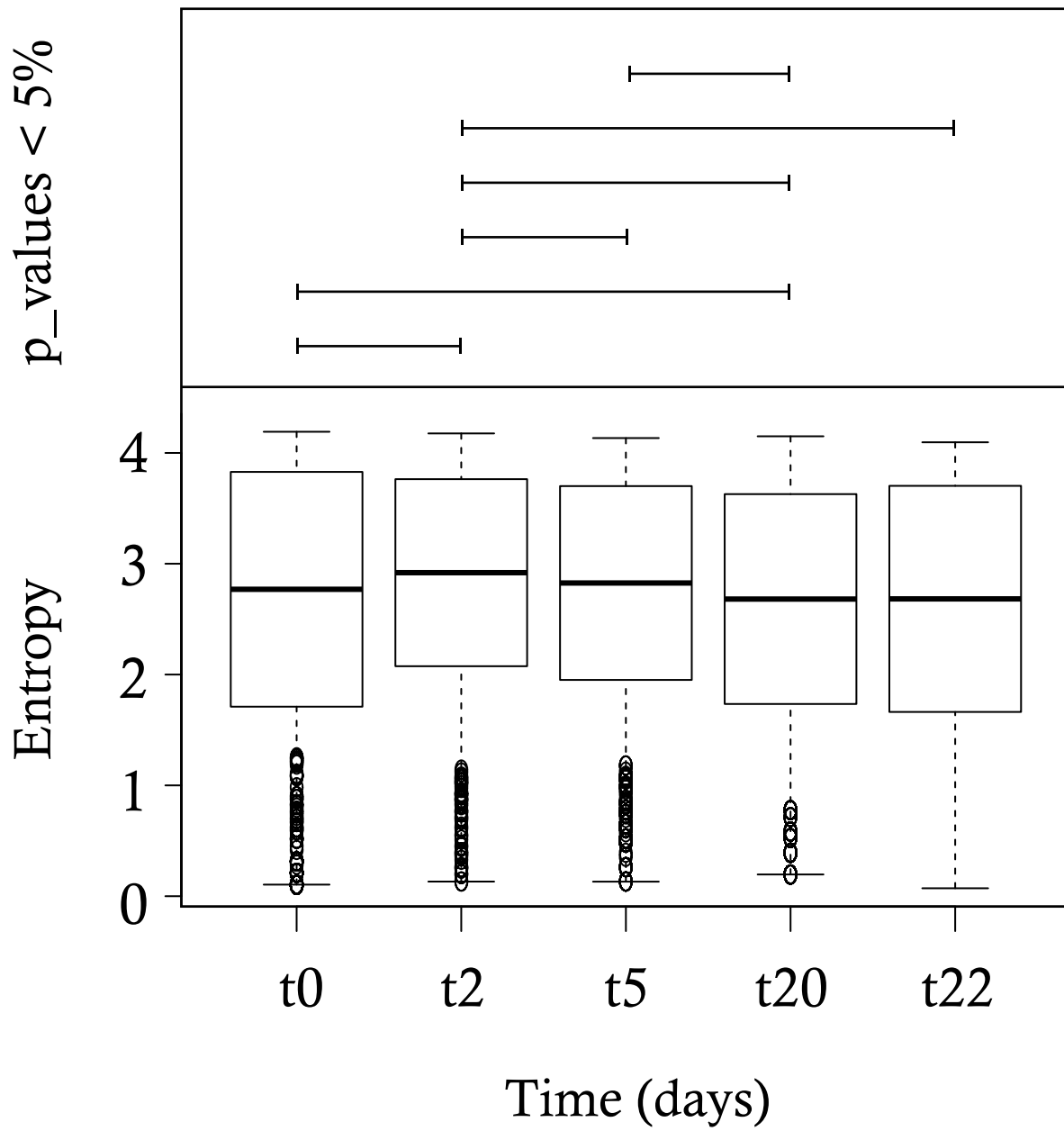

Supplement: S8 Fig — We computed differential gene expression between 0 and 2 d using the scRNA-seq data from [58]. We then computed an entropy value per time-point for the 776 resulting genes. Statistical significance was computed using a Wilcoxon test. (PDF) [file pbio.1002585.s008.pdf]

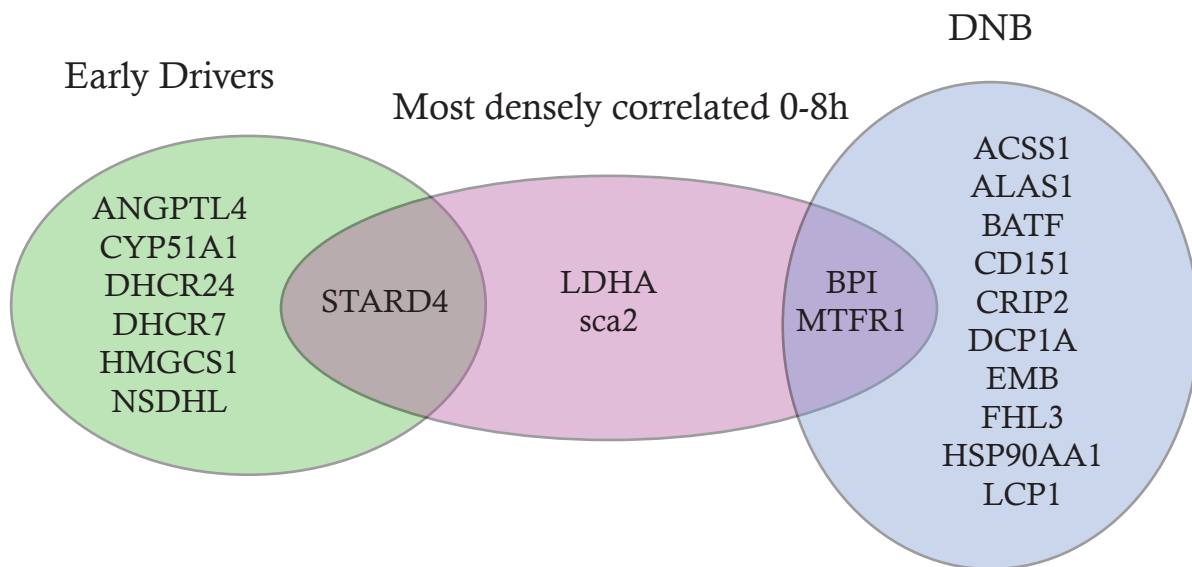

Supplement: S9 Fig — The Venn diagram shows the overlap of the three lists of genes obtained from the initial expression waves analysis (green), the correlation networks (pink), and the DNB theory (blue). The common genes between these lists were searched at 0 and 8 h when all three analyses have been performed (early driver genes were only identified between 0 and 8 h). (PDF) [file pbio.1002585.s009.pdf]
